# Synthesis methods impact silver nanoparticle properties and phenolic compound production in grapevine cell cultures

**DOI:** 10.1038/s41598-025-85545-7

**Published:** 2025-03-05

**Authors:** Nilgün GöktürkBaydar, Zehra Babalik, Tunahan Demirci, Alper Cessur

**Affiliations:** 1 Department of Agricultural Biotechnology, Faculty of Agriculture, Isparta University of Applied Sciences, Isparta, 32270 Turkey; 2 Department of Plant and Animal Production, Atabey Vocational School, Isparta University of Applied Sciences, Atabey-Isparta, 32670 Turkey; 3 Department of Pharmaceutical Biotechnology, Faculty of Pharmacy, Suleyman Demirel University, Isparta, 32260 Turkey

**Keywords:** Silver nanoparticles, Green synthesis, Kalecik Karası, Cell suspension cultures, Phenolic compounds, Nanoparticles, Secondary metabolism, Plant physiology

## Abstract

Silver nanoparticles (AgNPs) are one of nanoparticles with promising applications in various fields due to their unique characteristics. This study was carried out to determine the effects of AgNPs obtained by different green syntheses procedures on their characteristic properties and the accumulation of phenolic compounds in cell suspension cultures of Kalecik Karası grape cultivar. AgNPs were obtained by 24 different green synthesis methods including modifications in extraction method, reaction pH and conditions. When the results of the analyses conducted to determine the structural properties of AgNPs are evaluated, it was observed that more spherical and smaller nanoparticles were synthesized under alkaline conditions. The smallest NP size was detected as 8.9 nm in NP11, while the largest NP size (59.6 nm) was found in NP19. AgNPs obtained at room conditions for 4 h and pH 7 significantly increased the total phenolic, *trans*-resveratrol, catechin and epicatechin contents, while water or methanol extracts used in the synthesis had no significant effect. As a result of the study, it was determined that not only the characteristic properties of AgNPs but also their effectiveness on the secondary metabolite production varied significantly depending on the extraction method, pH and conditions of the reaction solution during synthesis.

**Supplementary Information:**

The online version contains supplementary material available at 10.1038/s41598-025-85545-7.

## Introduction

Nanoparticles (NPs) ranging in size from 1 to 100 nm exhibit improved catalytic reactivity, thermal conductivity, nonlinear optical performance, biological activity, and chemical stability compared to their macro forms due to their large surface area/volume ratio^1^. These properties have enabled nanoscale particles to be actively used in many fields such as medicine, biotechnology, environment, energy, textile, electronics, military applications, computing, space science and many more^2^. Although physical and chemical technologies allow the synthesis of NPs with high solubility and desired dimensions in a short time, they have some disadvantages such as high toxic content, low particle stability, and requirement for expensive technologies^3^. So the eco-friendly, cheap, and easy synthesis method called “green synthesis”, in which NPs are obtained using living structures, has gained great popularity as an alternative synthesis method^4^. Green synthesis involves the use of plants and microorganisms, and the method is based on a redox reaction in which metal ions are reduced to stable NPs by components of the microorganism or plants. NPs produced through green synthesis generally have better bioactivities and catalytic properties compared to those synthesized by physical and chemical methods^5^, and this is thought to be a result of the compounds added to their surfaces^1^.

Metal NPs such as silver, nickel, gold, palladium, copper and iron have been successfully obtained by green synthesis^3,5,6^. Microorganisms such as algae, fungi, and bacteria as well as plants and or plant wastes can be used in the synthesis of NPs. However, synthesis using plants or plant wastes is more advantageous than synthesis using microorganisms due to their simplicity, low cost, environmentally friendly properties, stable structures, and easy handling^1^. Therefore, plant extracts obtained from different plant parts and wastes can be successfully used in the synthesis of NPs^3,4,6–11^. Plant extracts can act as both reducing and stabilizing agents in the synthesis of NPs and significantly affect the properties of the NPs^7^. Grapevine leaves are one of the plant materials that can be successfully used in NP biosynthesis due to their rich phenolic content^6,12,13^. Because of their high tendency to chelate metals, phenolic compounds with hydroxyl and carboxyl groups show high antioxidant activity. Therefore, it has been determined that plants with high phenolic content are among the best candidates for NP synthesis^8^. Indeed, grapevine rich in phenolic contents can be successfully used as a plant material for obtaining NPs showing high bioactive effects^6,12,13^.

Silver nanoparticles (AgNPs) are the most commonly used NPs compared to other metal particles due to their antimicrobial effects^4,13–15^. In addition to antimicrobial effects, AgNPs are among the next-generation applications that can be used to increase secondary metabolite production under in vitro conditions^16–19^. In vitro secondary metabolite production is a culture technique that enables the production of very valuable metabolites used in different fields such as medicine, food, cosmetics, and perfumery in stable quality and quantity at all times of the year, away from climatic and geographical restrictions^20^. In this technique, where metabolite production is achieved by culturing plant parts such as roots, cells, shoots, and calli in sterile nutrient media and under controlled conditions, it was possible to increase metabolite production with external applications. Modifications in nutrient media and cultural conditions, precursor applications, immobilization, and elicitors that stimulate metabolite production by acting as signaling molecules are among the applications that can be used in this context^21^. One of the applications that can be used to increase metabolite yield in in vitro conditions is AgNP applications. Indeed, several studies have shown that AgNPs can increase the synthesis of secondary metabolites such as alkaloids and phenolics in various types of plant cultures belonging to different plants^16–19,22^. However, besides the plant material used in the synthesis of AgNPs, the extraction method and the reaction conditions such as pH, temperature and duration of the reaction in which the plant extract and silver nitrate (AgNO_3_) are combined, significantly change both the characteristics and the bioactivities of the AgNPs^6–8,23^.

This study aimed to investigate the characteristic properties of 24 different AgNPs obtained using various synthesis methods and their effects on growth criteria and the accumulation of phenolic compounds in grape cell suspension cultures to determine the most suitable NP synthesis method in terms of these criteria.

## Materials and methods

### Green synthesis of AgNPs

Leaves of *Vitis vinifera* L. cv Kalecik Karası (red wine grape cultivar) were used in this study as plant materials. The dried and powdered leaves were extracted using four different methods, as detailed in Table 1, to obtain the leaf extracts for use in the green synthesis of AgNPs.

**Table 1:** Methods used to obtain leaf extracts.

Extraction code	Extraction methods
E1	Boiling a mixture of leaf sample and methanol (1:6, w/v) for 5 min followed by stirring at room temperature for 1 h
E2	Boiling a mixture of leaf sample and distilled water (1:6, w/v) for 5 min followed by stirring at room temperature for 1 h
E3	Mixing a combination of leaf sample and methanol (1:6, w/v) at room temperature for 1 h
E4	Mixing a combination of leaf sample and distilled water (1:6, w/v) at room temperature for 1 h

**Fig. 1:**
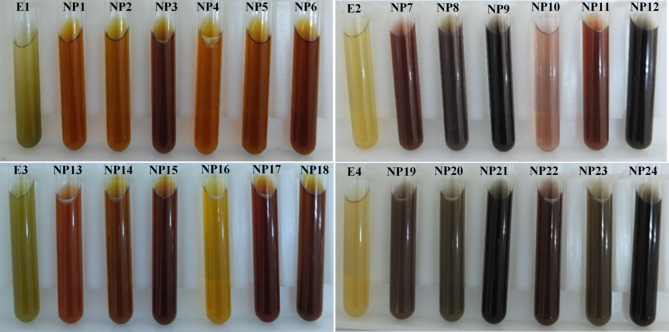
Colour changes in the reactions as a result of different extraction and green synthesis applications (E1, E2, E3, E4, plant extracts obtained by different extraction methods; NP1-NP24, AgNPs (The explanations of abbreviated variables are given in Tables 1 and 3).

After the processes, the extracts were filtered through coarse and Whatman No.1 filter papers, respectively, to remove any solid particles or impurities. In order to obtain AgNPs by green synthesis, 10 ml of each of these extracts was first mixed with 90 ml of 1 mM silver nitrate (AgNO_3_) solution^11^, and then these mixtures were subjected to 6 different treatments involving modifications in pH and the reaction procedure (Table 2). Thus, 24 different AgNPs were obtained using 4 extraction and 6 synthesis methods (Table 3; Fig. 1).

**Table 2:** Methods used for green synthesis of AgNPs.

Method code	pH	Reaction procedure
U1	4	Stirring at 60 °C for 20 min followed by 4 h at room temperature
U2	7	Stirring at 60 °C for 20 min followed by 4 h at room temperature
U3	10	Stirring at 60 °C for 20 min followed by 4 h at room temperature
U4	4	Stirring for 4 h at room temperature
U5	7	Stirring for 4 h at room temperature
U6	10	Stirring for 4 h at room temperature

**Table 3:** Codes of AgNPs obtained by different extraction methods and synthesis procedures.

AgNP code	Extraction method	Synthesis method	AgNPs	Extraction method	Synthesis method
NP1	E1	U1	NP13	E3	U1
NP2	E1	U2	NP14	E3	U2
NP3	E1	U3	NP15	E3	U3
NP4	E1	U4	NP16	E3	U4
NP5	E1	U5	NP17	E3	U5
NP6	E1	U6	NP18	E3	U6
NP7	E2	U1	NP19	E4	U1
NP8	E2	U2	NP20	E4	U2
NP9	E2	U3	NP21	E4	U3
NP10	E2	U4	NP22	E4	U4
NP11	E2	U5	NP23	E4	U5
NP12	E2	U6	NP24	E4	U6

After the synthesis processes were completed, UV-Vis analysis was performed to determine the conversion of silver ions to AgNPs. Subsequently, the mixtures were centrifuged at 9000 rpm for 20 min. AgNPs that gathered at the bottom of the centrifuge tube were separated from the supernatant and washed 2–3 times with distilled water. The AgNP pellets were then dried at 35^o^C to constant weight and prepared for further analyses.

### Characterization of AgNPs

UV-Vis analyses were initially performed to determine the conversion of silver ions into AgNPs. For this purpose, after the synthesis process, 0.2 ml of each of the plant-AgNO_3_ mixtures were dissolved in 2 ml distilled water using an ultrasonic water bath and their absorbances between 250 and 700 nm wavelengths were measured by UV-Vis spectrophotometry (PG instruments - T70 UV-Vis)^13^. The crystal phase composition of the AgNPs were determined by X-Ray Diffractometer (XRD) (Panalytical Empyrean) in the range of 10° ≤ 2θ ≥ 90° and the crystal sizes of AgNPs were calculated using the Debye-Scherrer formula given below^24^.

$${\text{D}}={\text{K}}\lambda /(\beta {\text{cos}}\theta )$$

D: crystalline size of NPs (nm), K: Scherrer constant (0.95), λ: the wavelength of the x-ray radiation (1.5406 Ao), β: the full width at half maximum (FWHM) of the intense and broad peaks; θ is the Bragg’s or diffraction angle.

Fourier transform infrared spectroscopy (FT-IR, Perkin Elmer Spectrum Two FT-IR and Pike Gladi ATR Instrument) was employed to identify the functional groups that the plant extract played a role in reducing while High-Resolution Transmission Electron Microscope (HR-TEM, Jeol Jem 1220 instrument) was used to provide a detailed visualization of the surface and fine structures of the NPs, to examine their morphological characteristics, and to interpret structure-function relationships. Dynamic light scattering (DLS, Horiba SZ -100 V2) analysis was used to characterize the size distribution of NPs, and zeta potential (Horiba SZ -100 V2) values were employed to determine the surface charge and push and pull forces of NPs in a colloidal solution.

### Obtaining and propagation of callus cultures

Before being transferred to the nutrient media, healthy petioles of Kalecik Karası grape cultivar were washed 3–5 times under running tap water, kept in 70% ethanol for 70 s, and then surface sterilized by shaking in 20% sodium hypochlorite solution for 15 min. After rinsing with sterile distilled water 3 times for 5 min each, the petioles cut into 1 cm pieces were transferred to nutrient media containing macro elements of B5 medium^25^ and micro elements and vitamins of MS medium^26^. Additionally 1 mg l^− 1^ benzylaminopurine (BAP), 0.1 mg l^− 1^ 2,4-dichlorophenoxy acetic acid (2,4-D), 30 g l^− 1^ sucrose, and 7 g l^− 1^ agar were added to media. Petiols were cultured at 25 ± 1 °C in the dark. The formed calli were then subcultured 3 times at 4-week intervals in the same nutrient media and culture conditions to propagate the calli.

### AgNP applications to cell suspension cultures

Cell suspension cultures were obtained by transferring approximately 2 g of calli into 50 ml of liquid media with the same content used for propagation of calli. Cell suspension cultures were cultured on a shaker at 90 rpm at 25 ± 1 °C in the dark for 15 days. Then 24 different AgNPs prepared in ultrapure water and kept in an ultrasonic water bath at room temperature for 4 h were added to the cell suspension cultures at a concentration of 8 mg l^− 1^. The control calli received only ultrapure water. Harvesting was performed on the 7th day after AgNP applications^27^. The harvested cells filtered through filter paper, were gently washed with sterile ultrapure water. After excess water was absorbed by blotting paper, they were used in the analyses. The research was set up in a randomized complete block design with three replications, each containing 5 Erlenmeyer flasks.

### Determination of the effects of AgNP applications on cell growth

Cell fresh weight (CFW) after harvest and cell dry weight (CDW) dried at 40 °C until constant weight were determined by weighing the cells on an analytical balance. The results are given in g 100 ml^− 1^. Cell growth index (GI) was also determined according to the following formula:

$${\text{GI }}={\text{ }}\left( {{\text{harvested CFW }}\left( {\text{g}} \right){\text{ }} - {\text{ inoculated CFW }}\left( {\text{g}} \right)} \right)/{\text{inoculated CFW }}\left( {\text{g}} \right)$$

### Extraction of phenolic compounds

The dried and powdered cells were extracted 3 times for 30 min in 70% methanol contained 0.1% hydrochloric acid (HCl) using an ultrasonic bath. The solvent was removed in a rotary evaporator under vacuum at 40 °C and dry extracts were dissolved in HPLC grade methanol and used in the analysis after filtering through 0.45 μm filter.

### Determination of total phenolic content

Total phenolic content in cells was determined using the Folin Ciocalteu method^28^. Absorbance readings of the samples were performed at 765 nm using a PG-170 UV-Vis spectrophotometer. The total phenolic contents of the samples were determined by using a curve of gallic acid equivalents (GAE) per gram of dried sample (mg GAE g^− 1^).

### Determination of phenolic compounds by HPLC

Phenolic compound analysis of cell extracts by HPLC was performed according to the method used by Göktürk Baydar et al.^29^. Amounts of gallic acid, catechin, epicatechin, *o*-coumaric acid, cinnamic acid, *p*-coumaric acid, *trans*-resveratrol, vanillin, ferulic acid, caffeic acid, chlorogenic acid, rutin, and quercetin were calculated as µg g^− 1^ in cells by comparing with the peak areas of the standards. The Shimadzu Class-VP Chromatography Laboratory Automatic Software System was used to calculate the results.

**Fig. 2:**
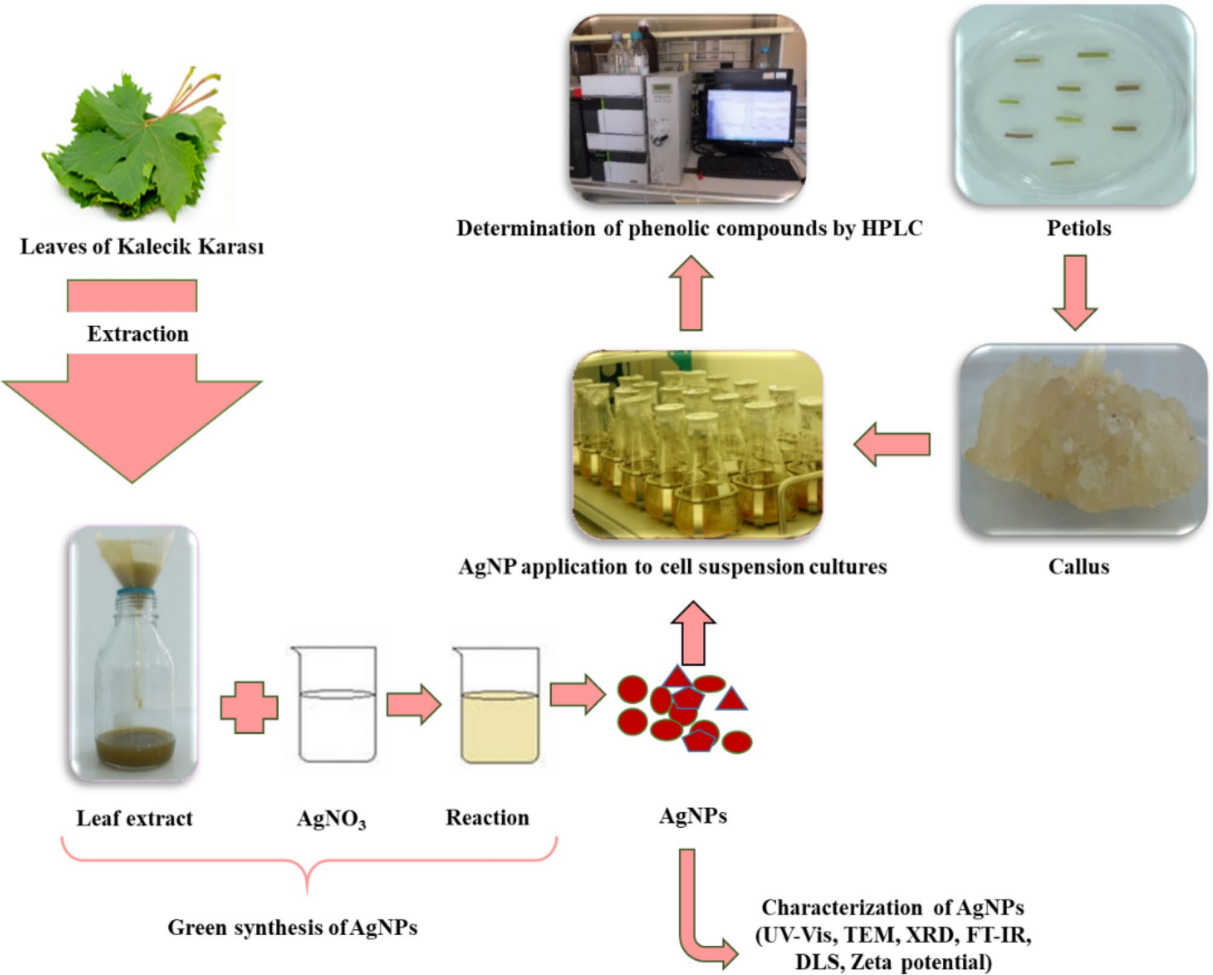
Schematic representation of the research.

The procedures performed in the research are given schematically in Fig. 2.

### Statistical analyses

SPSS 26.0 statistical program was used to evaluate the data obtained in the study and the differences between the treatments were determined by Duncan Multiple Comparison Test (*p* ≤ 0.05). Pearson correlations were performed in R using the RStudio V4.3.1 environment (R Studio Team 2023; R Core Team 2023, Vienna, Austria) with the corrplot package. Heat map analysis using the averages of the data and grouping of the data was performed with ClustVis web tool as described by Metsalu and Vilo^30^ .

## Results

**Fig. 3:**
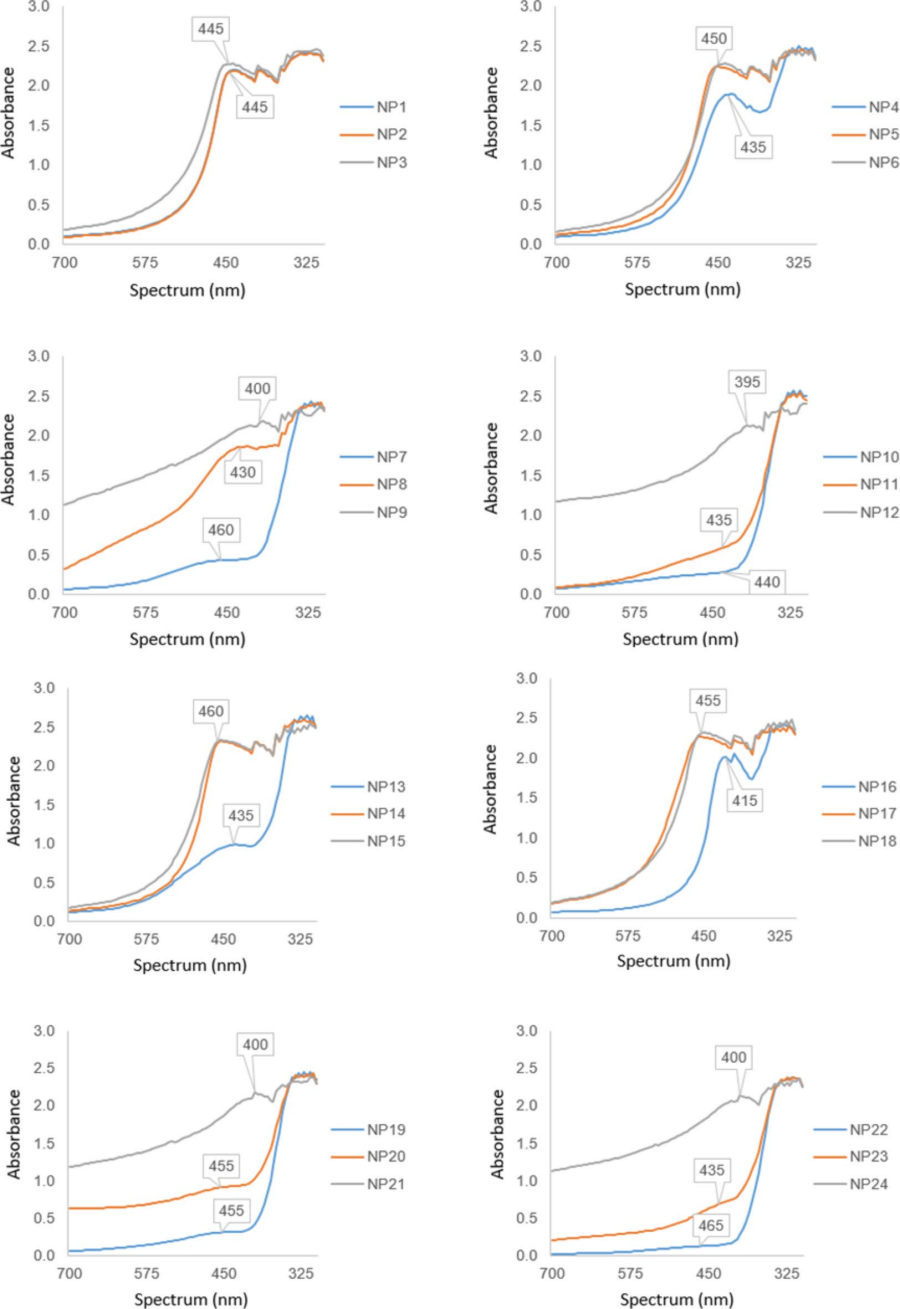
UV-Vis spectra of AgNPs.

Characterization analysis of AgNP is of great importance as it provides information about the shape, size, size distribution, surface charge and general physiochemical properties of NPs. In this study, UV-Vis, HR-TEM, XRD, FT-IR, DLS, and zeta potential analyses were done to determine the characterizations of AgNPs. According to UV-Vis results, the peaks of the spectra were found to be around 450 nm. The AgNPs synthesized using methanol extracts (NP1-NP6 and NP13-NP18) exhibited a broad and distinct peak in the range of 350 to 600 nm, with the peak maximum at approximately 450 nm (Fig. 3). In contrast, the AgNPs synthesized using water extracts (NP7-NP12 and NP19-NP24) displayed less distinct peaks and showed absorbance in the range of 300 to 450 nm. Except for the solvent, the extraction methods made no significant difference in the UV-Vis spectra. Compared to AgNPs synthesis at pH 4, those synthesized at pH 7 and pH 10 showed more intense absorbance and their peaks between 300 and 600 nm are consistent with the absorbance of typical AgNPs. While pH did not make a significant difference in the applications using methanol extractions (NP1-NP6 and NP13-NP18), the UV absorbance was lower at pH 4 than at pH 7 and pH 10 in the applications using water extractions (NP7-NP12 and NP19-NP24). The absorbance analysis by UV-Vis clearly showed that the synthesized particles were AgNPs, although the extraction methods and reaction pH and conditions caused changes in the absorbance values of AgNPs and the peaks they formed.

**Fig. 4:**
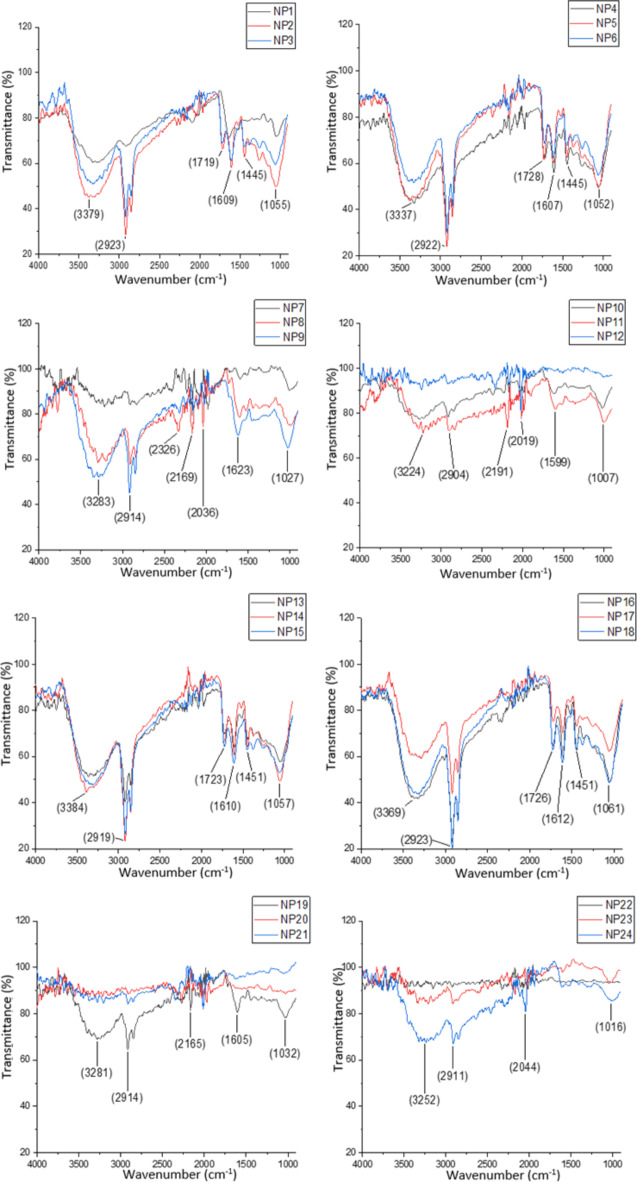
FT-IR spectra of AgNPs.

The vibrational intensities of the prominent peaks in the FT-IR spectra of AgNPs in the ranges of 3200–3500 cm^− 1^, 3000–2800 cm^− 1^, 2400–2000 cm^− 1^, 1800–1600 cm^− 1^, 1450–1350 cm^− 1^, and 1070 –1000 cm^− 1^ varied depending on the extraction methods, pH and the reaction conditions (Fig. 4). Under the same reaction conditions, AgNPs synthesized with methanol-based extracts (NP1-NP6 and NP13-NP18) exhibited more distinct and functional groups in their FT-IR spectra compared to those synthesized with water-based extracts (NP7-NP12 and NP19-NP24). The FT-IR spectra of AgNPs synthesized under different pH and reaction conditions using methanol extracts exhibited a high degree of similarity while there were significant differences between the FT-IR spectra of AgNPs synthesized with the U1 and U2 methods using water extracts.

**Fig. 5:**
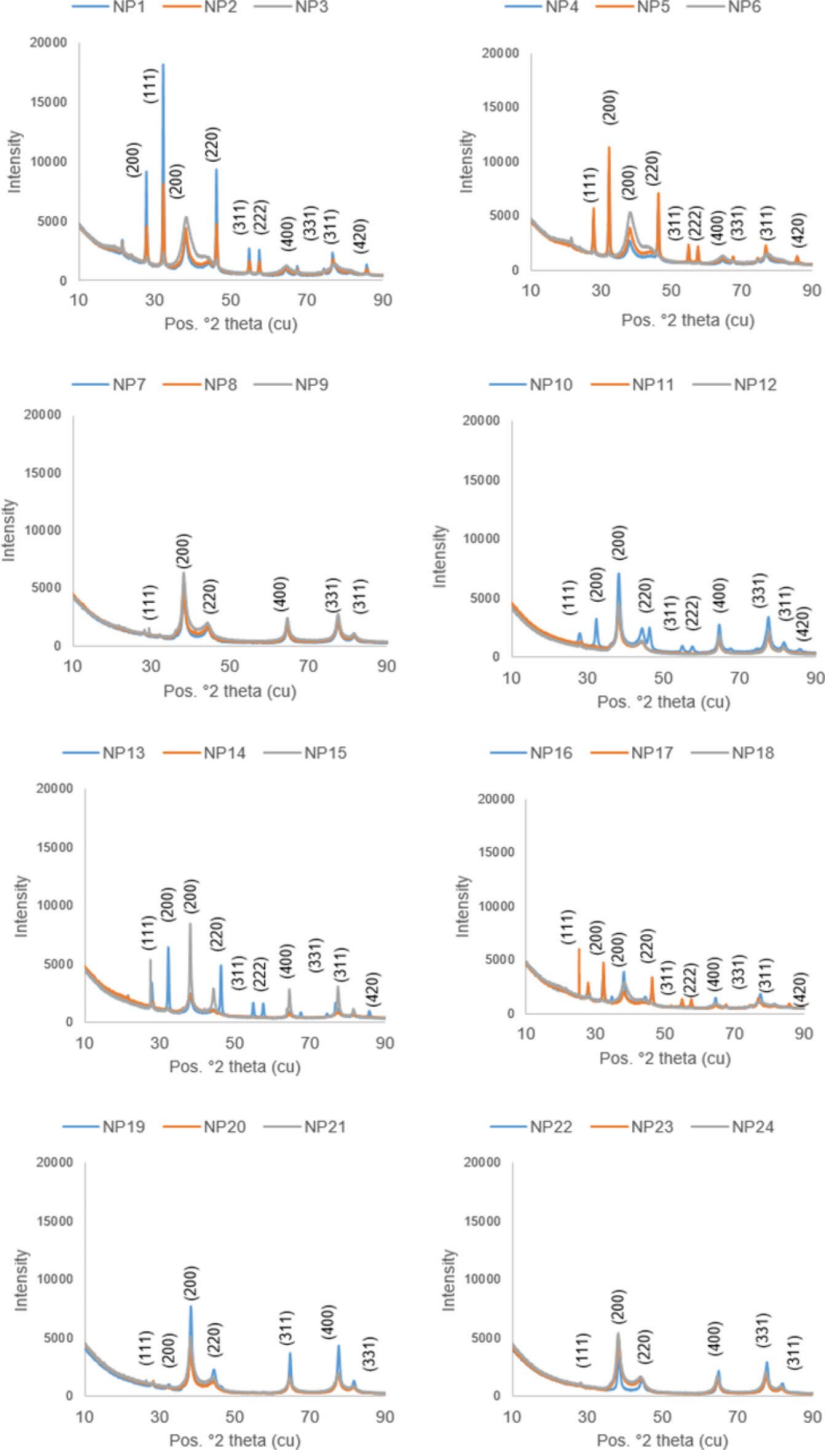
XR diffractograms of AgNPs.

XRD analyses showed peaks at 2 theta values between 10° and 90° depending on the AgNPs. Within the XRD model, prominent peaks manifested at 2θ values of 28, 32, 38, 46, 55, 66, and 75. Diffraction corresponding to these peaks was observed at levels (111), (200), (220), (400) and (331) (Fig. 5). When the XRD results were analyzed with the Match program (Crystal impact, Bonn, Germany) database, they were highly matched with the diffractions formed as a result of the reduction of Ag + cation. AgNPs except for NP7, NP12, NP18, and NP19 with triangular, cube, and multilateral shapes were in a spherical form. AgNPs with triangular, cube, and multilateral shapes were obtained from applications where the pH of the reaction solution was adjusted to 4 and 10. AgNPs synthesized with E1 extract (NP1-NP6) had more intense peaks compared to those of E3 extract (NP13-NP18). It was also found that AgNPs synthesized by using methanol extract (NP1-NP6 and NP13-NP18) had more and more intense peaks than AgNPs synthesized using water extract (NP7-NP12 and NP19-NP24).

The average particle sizes of AgNPs calculated using the Debye-Scherrer equation, based on the observed peaks in the XRD graphs, showed significant variations depending on different synthesis methods (Table 4). The largest average particle size was obtained from NP19 with 59.6 nm, while the smallest average particle size was obtained from NP11 with 8.9 nm.

**Table 4:** The average particle sizes of AgNPs calculated by the Debye-Scherrer equation.

AgNPs	Average particle size (nm)	AgNPs	Average particle size (nm)
NP1	26.7	NP13	25.4
NP2	39.3	NP14	33.5
NP3	18.6	NP15	18.5
NP4	27.8	NP16	28.6
NP5	17.8	NP17	24.5
NP6	31.2	NP18	38.6
NP7	26.4	NP19	59.6
NP8	23.7	NP20	12.7
NP9	22.6	NP21	39.4
NP10	18.9	NP22	21.4
NP11	8.9	NP23	27.4
NP12	35.1	NP24	18.7

**Fig. 6:**
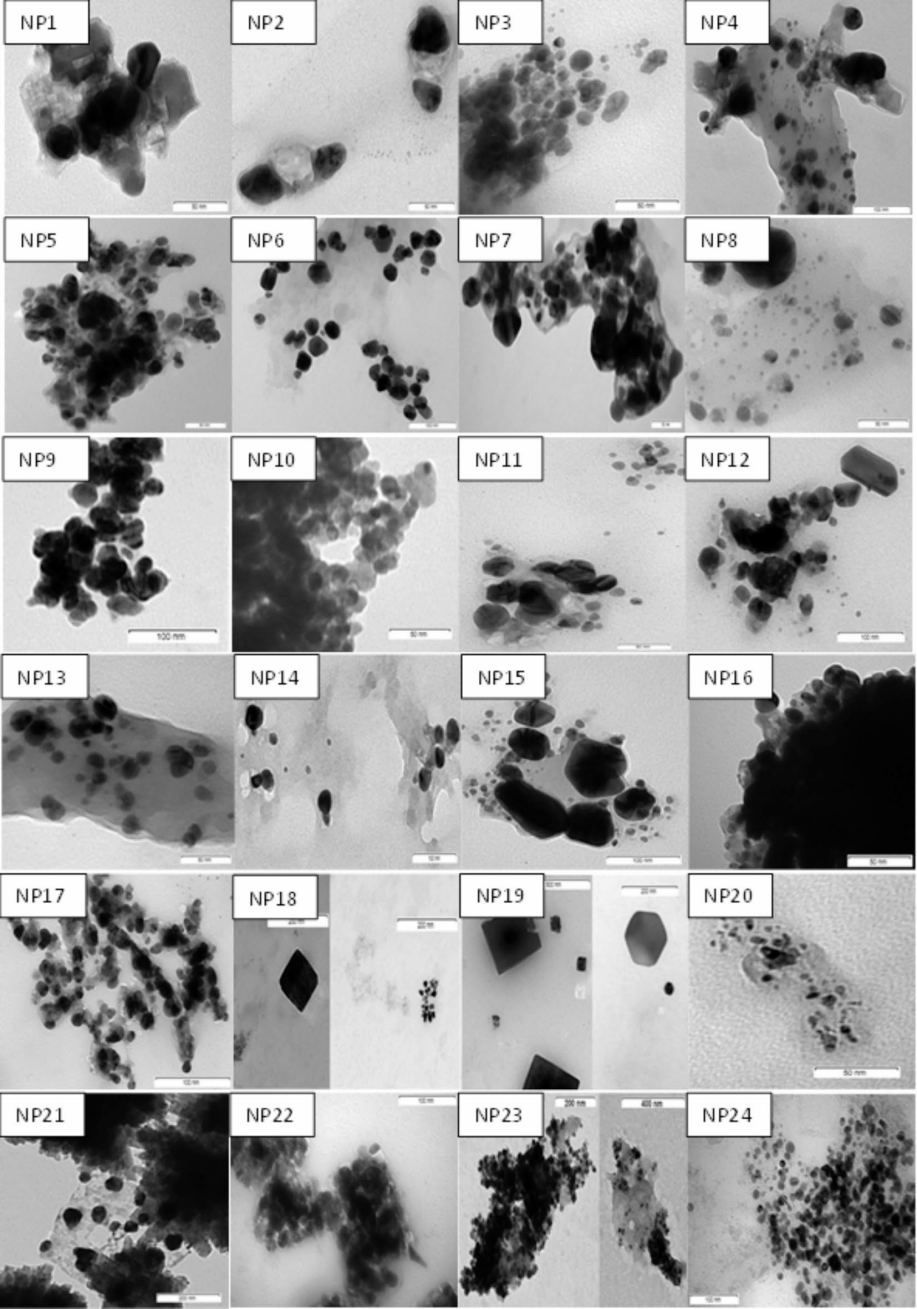
HR-TEM images of AgNPs.

When the HR-TEM images of AgNPs were examined, it was found that AgNPs generally have a spherical shape, rarely multilateral triangular or cube shape (NP7, NP12, NP18, NP19) (Fig. 6). The pH affected the size and shape of AgNPs. In general, cubic and multilateral AgNPs were synthesized at pH 4 and pH 10. AgNPs mostly did not aggregate uniformly, but some AgNPs formed small groups. NP10, NP16, NP22 and NP23 were aggregated and could not be homogenized during HR-TEM imaging. AgNPs synthesized at pH 7 using methanol extract (NP2, NP5, NP14, and NP17) were found to be less aggregated and AgNPs were well homogenized during HR-TEM; at pH 4 and 10 they were found in small groups attached to each other with electron cloud. Generally, it was observed that AgNPs tended to be smaller and more spherical as the pH increased, but variations were noted depending on the extraction and reaction conditions.

**Fig. 7:**
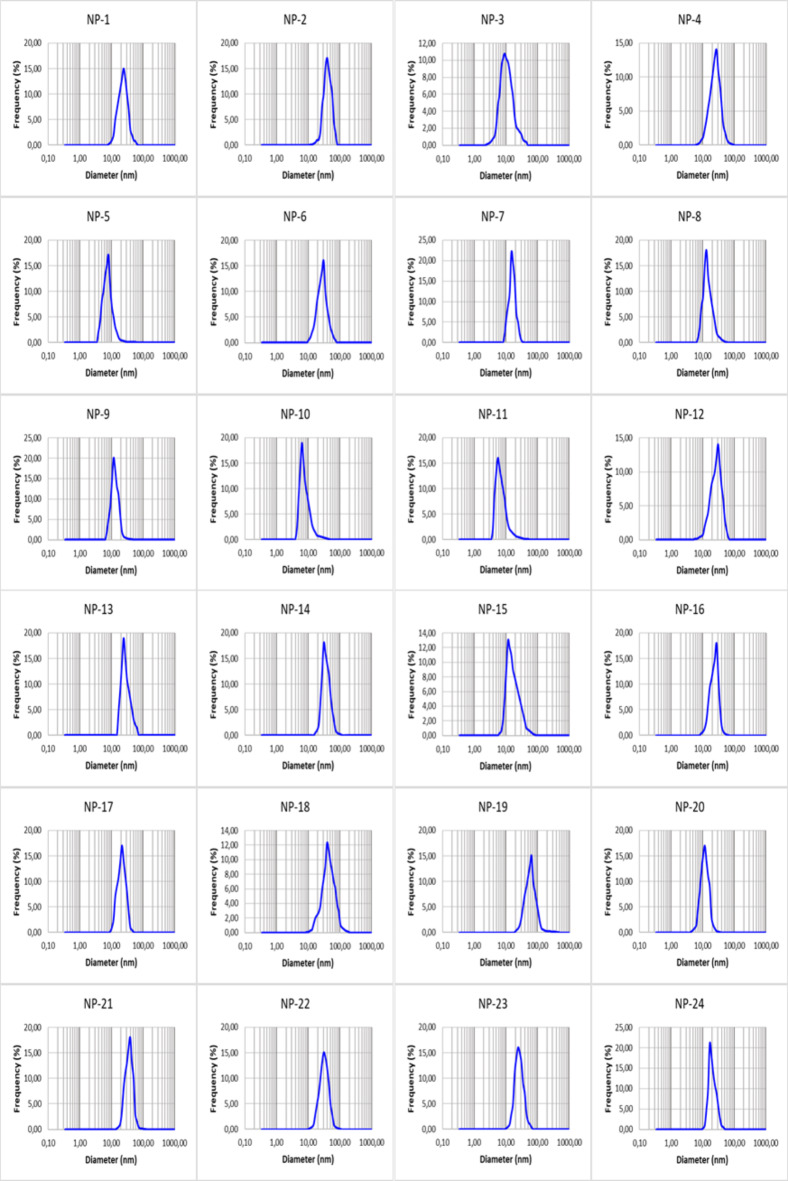
DLS analyzes of the particle sizes distribution in AgNPs.

DLS analyses showed that particle sizes varied depending on the extraction method, pH and the reaction conditions (Fig. 7). AgNPs were synthesized with sizes ranging from 5  to 500 nm, but their average size varied between 8 and 60 nm. These results are in agreement with the average particle size data calculated by the Debye-Scherrer equation.

The zeta potentials of AgNPs were greater than + 30 mV and less than − 30 mV, indicating a stable structure (Table 5). However, NP7, NP10, NP16, NP22, and NP23 were found to have zeta potentials of − 29, − 20, − 18, − 25, and − 29 mV, respectively, showing a less stable structure and aggregating with each other. These results are in parallel with the imaging results obtained from HR-TEM microscopy. Zeta potential images were submitted in supplementary materials.

**Table 5:** Zeta potentials of AgNPs.

AgNPs	Zeta potential (mV)	AgNPs	Zeta potential (mV)
NP1	− 67	NP13	− 78
NP2	− 83	NP14	− 55
NP3	− 69	NP15	− 39
NP4	− 45	NP16	− 18
NP5	− 49	NP17	− 63
NP6	− 41	NP18	− 51
NP7	− 29	NP19	− 46
NP8	− 45	NP20	− 25
NP9	− 46	NP21	− 47
NP10	− 20	NP22	− 29
NP11	− 47	NP23	− 42
NP12	− 56	NP24	− 89

### The effects of AgNPs on cell growth parameters

**Fig. 8:**
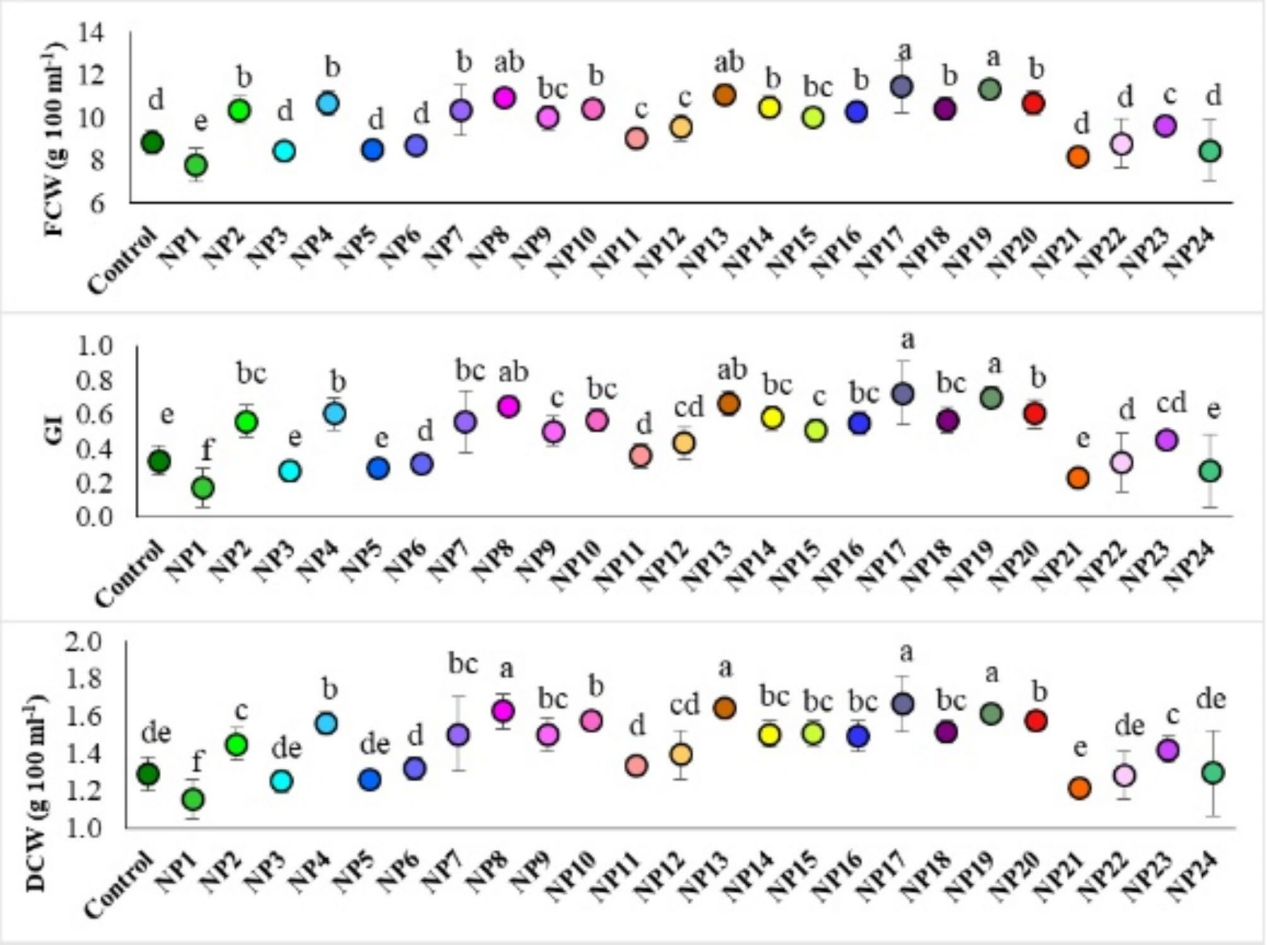
Effect of AgNPs on CFW, GI and CDW. Data represent the mean ± standard deviation. Different letters indicate statistically significant differences among the treatments (*p* ≤ 0.05, Duncan’s test).

The effects of AgNPs synthesized by different methods on growth parameters, including CFW, GI and CDW, varied significantly as a result of applications (*p* ≤ 0.05) in grapevine cell suspension cultures (Fig. 8). The highest values of CFW and GI were obtained from the NP17 and NP19, followed by NP13 and NP8. The N17 and N19 resulted in a 1.29- and 1.27-fold increase in CFW, also a 2.18- and 2.12-fold increase in GI, respectively, compared to the control. On the other hand, the NP1 had an inhibitory effect on CFW and GI. The highest CDW was obtained from NP8 (1.62 g 100 ml^− 1^), NP13 (1.64 g 100 ml^− 1^), NP17 (1.67 g 100 ml^− 1^) and NP19 (1.61 g 100 ml^− 1^), while the lowest CDW was determined in NP1 with 1.15 g 100 ml^− 1^.

### The effect of AgNPs on the production of phenolic compounds

**Fig. 9:**
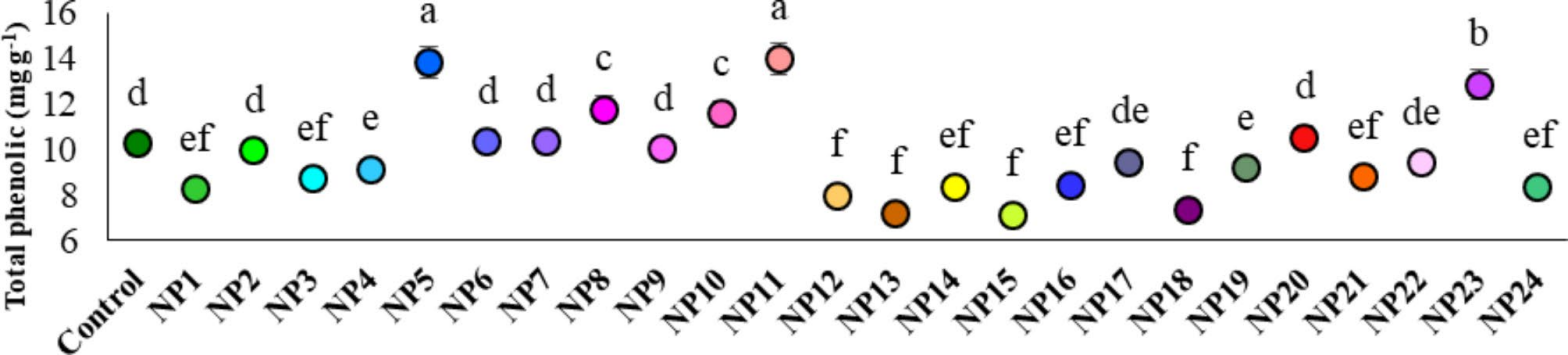
Effect of AgNPs on total phenolic contents of cells. Data represent the mean ± standard deviation. Different letters indicate statistically significant differences among the treatments (*p* ≤ 0.05, Duncan’s test).

The effects of AgNPs synthesized under different pH and reaction conditions using extracts obtained from grapevine leaves by different methods on the total phenolic content of cells are presented in Fig. 9. In the study, the total phenolic content of the cells varied significantly (p ≤ 0.05) according to the AgNPs synthesized by different methods. The highest total phenolic content was obtained from NP11 and NP5 with very close values of 13.98 mg g^− 1^ and 13.86 mg g^− 1^, respectively. Another remarkable result in the study was that AgNPs synthesized from all extracts adjusted to pH 7 (NP2, NP5, NP8, NP11, NP14, NP17, NP20, and NP23) showed a greater increase in total phenolic content compared to applications where pH was adjusted to 4 and 10.

**Fig. 10:**
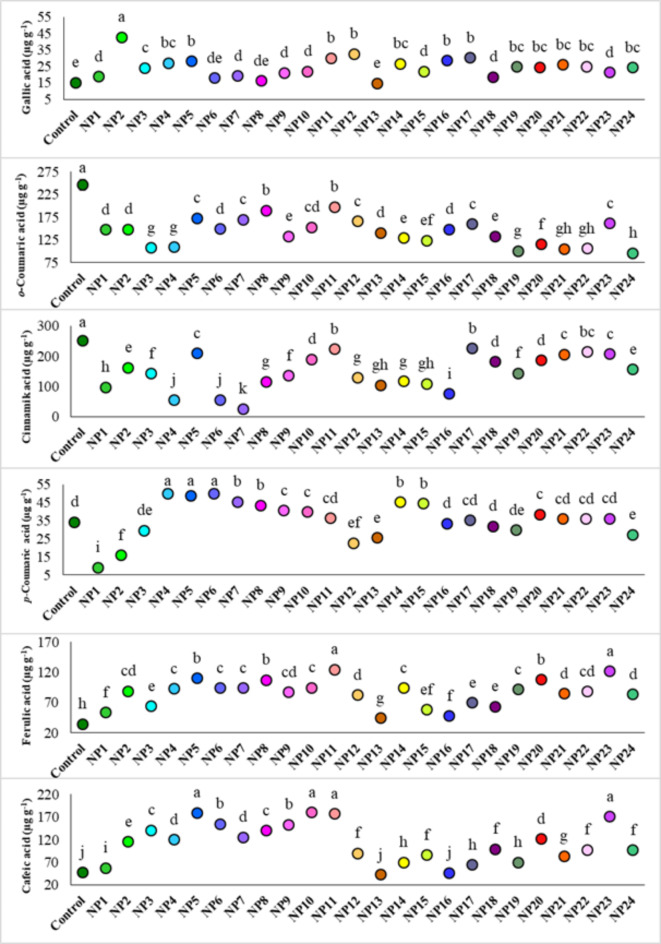
Effect of AgNPs on phenolic acids of cells. Data represent the mean ± standard deviation. Different letters indicate statistically significant differences among the treatments (*p* ≤ 0.05, Duncan’s test).

In this study, changes in phenolic acids in grapevine cell cultures depending on AgNP applications were also investigated (Fig. 10). For this purpose, gallic acid, cinnamic acid, *p-*coumaric acid, *o-*coumaric acid, ferulic acid, cafeic acid, and chlorogenic acid were determined in the cells. Chlorogenic acid could not be detected in cultures while amounts of other phenolic acids changed significantly according to AgNP applications (*p* ≤ 0.05). In terms of gallic acid, with 42.57 µg g^− 1^ the highest value was obtained from the cells treated with NP2. All AgNP applications except NP8 and NP13 caused higher gallic acid accumulation comparasion with the control, while the amounts of *o-*coumaric acid and cinnamic acid decreased significantly with AgNP applications compared to the control. The effects of AgNP applications on *p-*coumaric acid accumulation varied depending on the extraction method and reaction conditions, while pH had no significant influence on this variation. NP4, NP5 and NP6 were the most affecting applications in terms of *p*-coumaric acid. The highest amount of ferulic acid was found in cells applied with NP11 with 124.32 µg g^− 1^ and NP23 with 121.94 µg g^− 1^ while the maximum caffeic acid values were obtained from the NP5, NP10, NP11 and NP23.

**Fig. 11:**
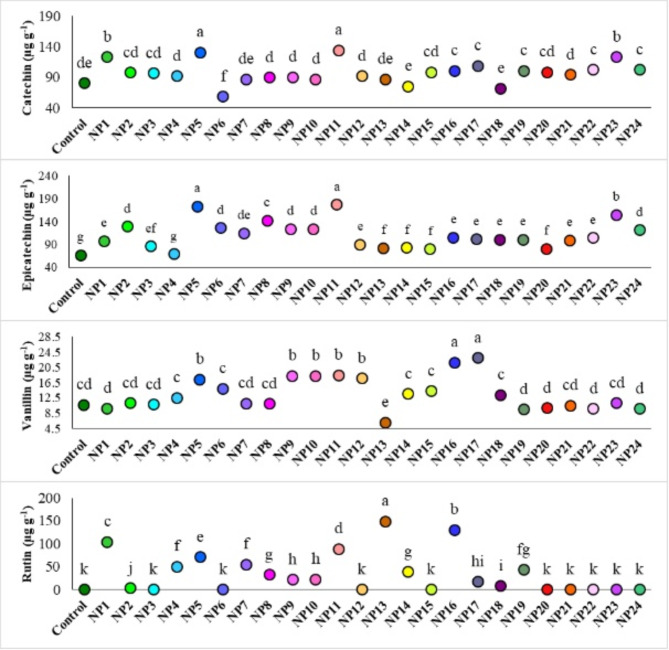
Effect of AgNPs on flavonoids of cells. Data represent the mean ± standard deviation. Different letters indicate statistically significant differences among the treatments (*p* ≤ 0.05, Duncan’s test).

The amounts of catechin, epicatechin, vanillin, rutin and quercetin as flavonoids in the cells were also analyzed (Fig. 11). While quercetin could not be detected in the cells, the amounts of other flavonoid compounds were found to vary significantly according to the AgNPs (*p* ≤ 0.05). Based on the results, the cells treated with NP11 and NP5 exhibited the highest levels of catechin (134.12 µg g^− 1^ and 130.82 µg g^− 1^, respectively) and epicatechin (176.32 µg g^− 1^ and 172.43 µg g^− 1^, respectively). The utilization of NP11 and NP5 resulted in the enhancement of both catechin and epicatechin accumulation in grapevine cells. The maximum values of vanillin were obtained from NP16 and NP17. Rutin was not detected in the cells of the control group as well as in cells treated with NP3, NP6, NP12, NP15, NP18, NP20, NP21, NP22, NP23 and NP24 while the greatest rutin values were detected in the cells treated with NP13 and NP16.

**Fig. 12:**
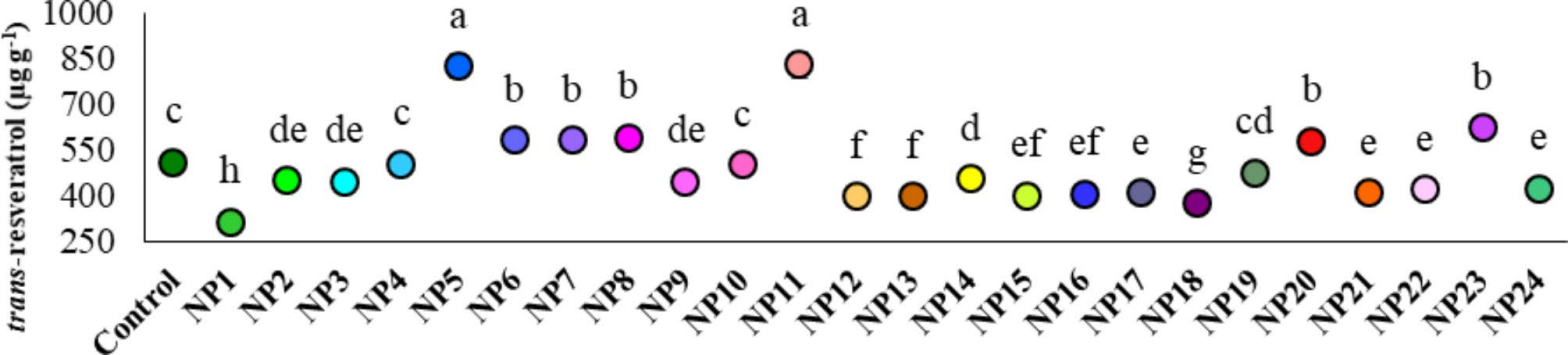
Effect of AgNPs on *trans-*resveratrol of cells. Data represent the mean ± standard deviation. Different letters indicate statistically significant differences among the treatments (p ≤ 0.05, Duncan’s test).

Significant alterations in *trans-*resveratrol accumulation within grapevine cell suspension cultures were observed after a 7-day application of AgNPs (*p* ≤ 0.05). Accordingly, the highest *trans-*resveratrol accumulations were obtained from the cells treated with NP11 and NP5 (830.19 µg g^− 1^ and 825.67 µg g^− 1^, respectively) as in catechin and epicatechin amounts. The cells treated with NP1 showed the lowest *trans*-resveratrol accumulation, with a value of 314.78 µg g^− 1^ (Fig. 12).

**Fig. 13:**
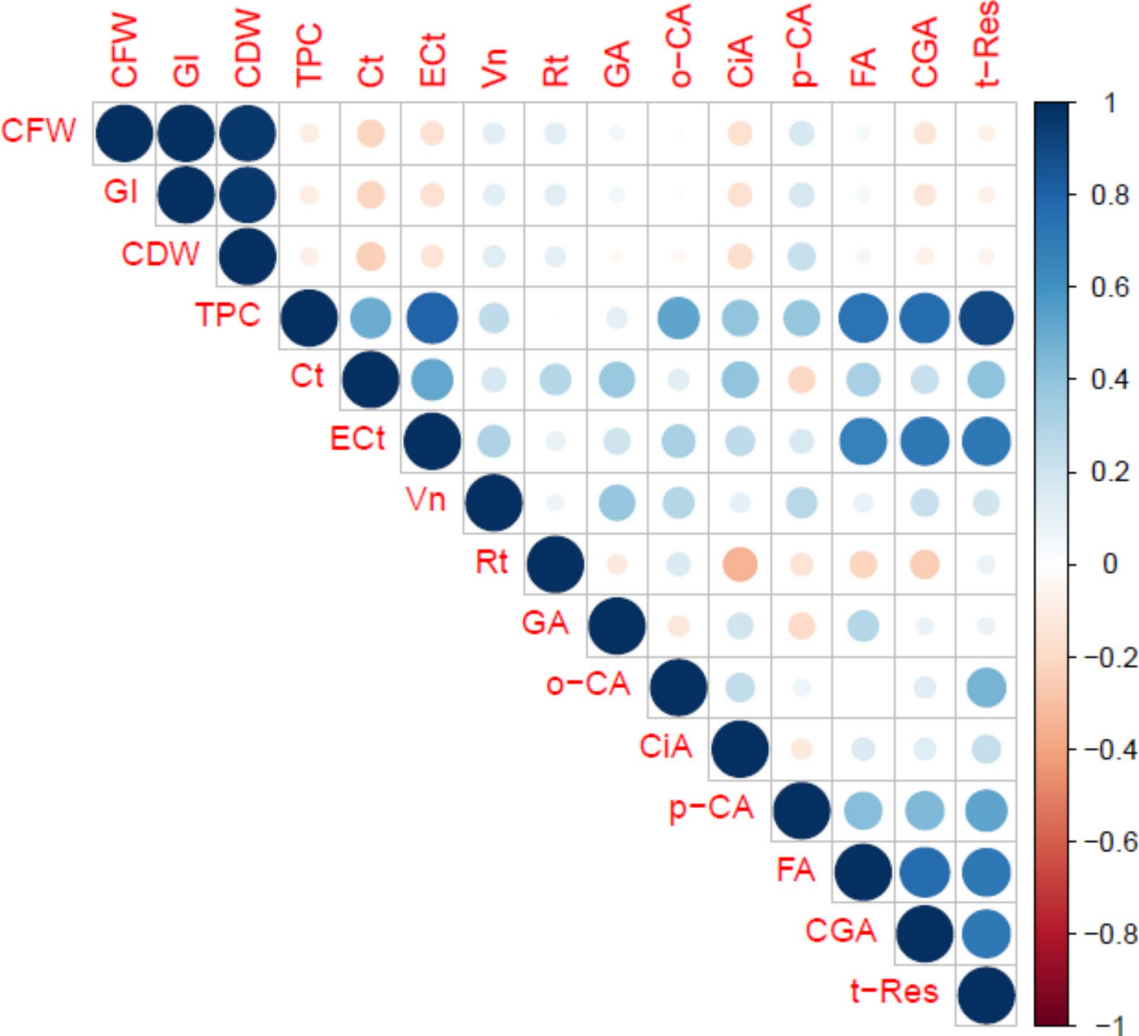
The correlation matrix of cell growth parameters and secondary metabolites. The matrix illustrates the strength of the Pearson correlation, with the shape and color of each dot in the triangular matrix indicating the magnitude and direction (positive or negative) of the correlation between the cell growth and secondary metabolites parameters. Dark blue dots represent strong positive correlations, while lighter colors indicate weaker relationships between the parameters. The full names of abbreviated parameters; *CFW* cell fresh weight, *GI* growth index, *CDW* cell dry weight, *TPC* total phenolic content, *Ct* catechin, *ECt* epicatechin, *Vn* vanillin, *Rt* rutin, *GA* gallic acid, *o-CA o-*coumaric acid, *CiA* cinnamic acid, *p-CA p-*coumaric acid, *FA* ferulic acid, *CGA* caffeic acid, *t-Res trans-*Resveratrol.

A Pearson correlation matrix analysis was performed in Fig. 13. Concerning the correlation coefficients, the results showed significant positive and negative correlations for cell growth parameters and phenolic compounds. CFW had significant positive correlations with GI and CDW and negative correlations with total phenolic content, catechin, epicatechin, cinnamic acid, caffeic acid and *trans*-resveratrol. GI had significant positive correlations with CDW. There was a significant positive correlation between total phenolic content and other phenolics except rutin, catechin had a negative correlation with p-coumaric acid, epicatechin, vanillin, o-coumaric acid, p-coumaric acid, ferulic acid, and caffeic acid had positive correlations with the other phenolic components. *trans*-Resveratrol had significant positive correlations with examined all phenolics but significant negative correlations with cell growth parameters.

**Fig. 14:**
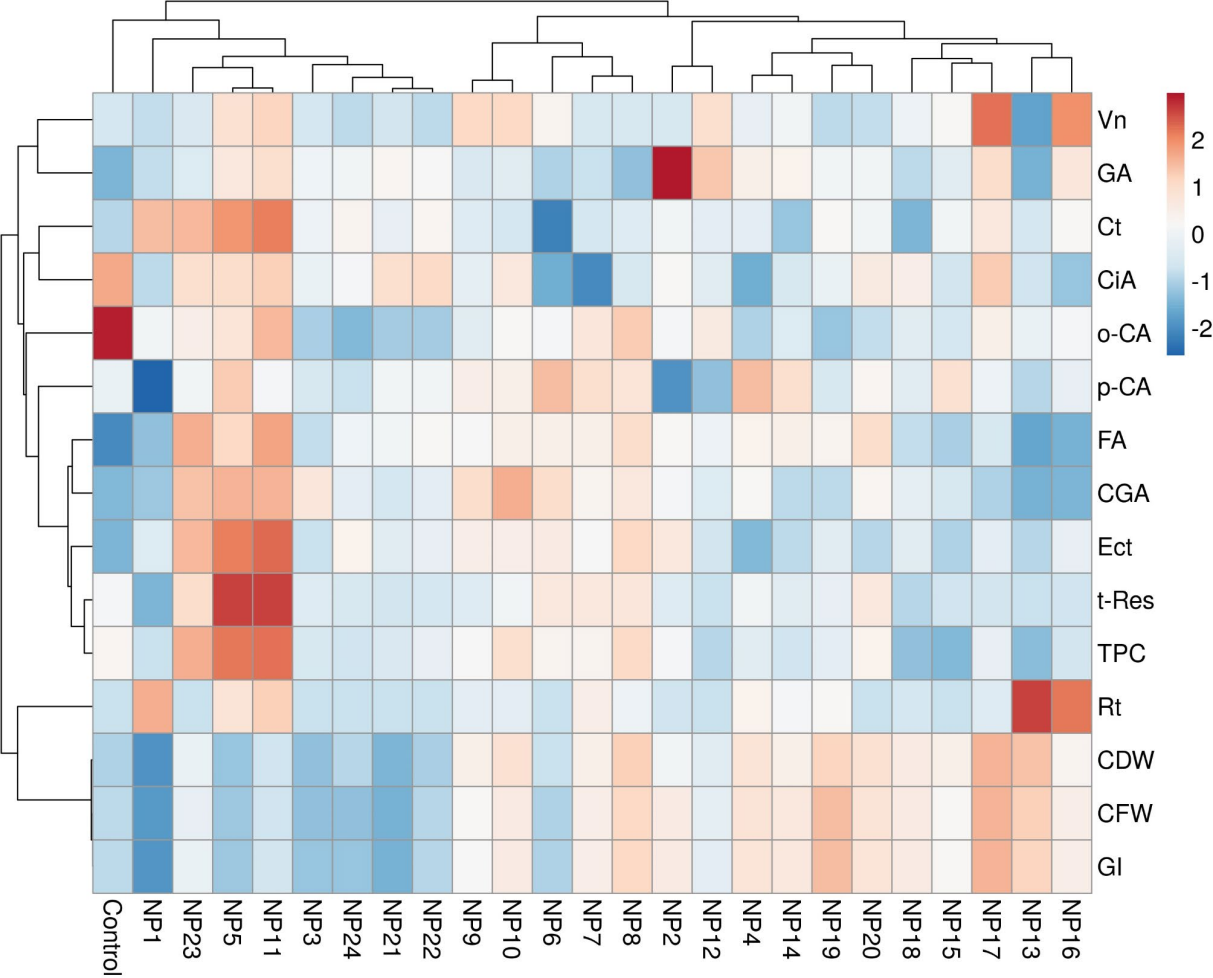
Heat map representing changes induced by applying AgNPs obtained through different synthesis methods to cell suspension cultures in growth and the accumulation of phenolic compounds.

In order to better understand the effect of AgNPs obtained from grapevine leaves by different synthesis methods on the cell growth and the accumulation of some important phenolic compounds, the data were visualized by heat map method and presented in Fig. 14. According to these results, cell growth parameters and contents of phenolic compounds of the cells were affected almost in opposite ways by AgNP treatments. It is evident from the densities of phenolic compounds and total phenolic content in the heat map that the accumulation of secondary metabolites was higher in the cells where growth parameters were low. Only the accumulations of rutin, gallic acid and vanillin were in parallel with the cell growth unlike other phenolic compounds. When AgNP treatments were evaluated within themselves, control, NP1, NP3, NP5, NP11, NP21, NP22, NP23, and NP24 treatments differed from the other AgNP treatments and did not significantly affect the growth but increased the accumulation of phenolic compounds. The treatments with high cell growth and phenolic accumulation were differentiated from other AgNP treatments as NP6, NP7, NP8, NP9, and NP10 in the heat map. When only phenolic compound accumulations were evaluated, it was observed that the most effective treatments in the heat maps were NP5 and NP11, followed by NP23. The lowest phenolic accumulations in the heat map were observed in NP13, NP15, NP16, NP17 and NP18.

## Discussion

Traditional NP synthesis methods such as chemical and physical have been utilized for years, but research has shown that green methods are more effective in producing NPs, offering advantages such as being environmental- friendly along with a reduced likelihood of failure, cost-effectiveness, and ease of use^3^. In green synthesis, extracts obtained from various plant species and their different parts such as leaves, seeds, roots, fruits, and isolated compounds can be successfully used^3,4,6,10,11^. Ponarulselvam et al.^31^ stated that the synthesis of AgNPs, especially from leaves, is a good source to produce stable NPs due to their metabolite content. Therefore, grapevine leaves rich in phenolic compounds were used in the synthesis of NPs in this study. AgNPs synthesized using plant extract show a yellowish-brown color due to the excitation of surface plasmon resonances in AgNPs^4,6,31^. Asaduzzaman et al.^13^ determined the formation of AgNPs in grape extracts by changing the color of the solution from white to brown and explained this transformation as occurring with the reduction of Ag^+^ ions to Ag^0^. In our study, the color changes from light yellow to dark brown in the reactions immediately after different extraction and green synthesis applications are indicative of the formation of AgNPs.

Characterization of NPs in terms of shape, particle size distribution, solubility, morphology, surface area, crystallinity, pore size, dispersibility in solution and aggregation behavior is very important to reveal the success of the NP synthesis method used^32^. The characterization encompasses a range of analytical methods such as UV-Vis spectroscopy, XRD, FTIR, TEM, DLS and zeta potential designed to investigate both the physical and chemical attributes of NPs^33^. UV-Vis spectra provide important information about the shape, size and distribution of AgNPs. Peaks observed at short wavelengths indicate the small size of AgNPs, while peaks at longer wavelengths indicate larger AgNPs^34^. Typically, the presence of peaks within the 420 to 430 nm range is indicative of the presence of AgNPs in the size range of 10 to 30 nm, exhibiting a spherical morphology^35^. Nevertheless, numerous studies have reported variations in the spectral ranges of AgNPs’ peaks. It has been suggested that these disparities can be attributed to fluctuations in pH, temperature, and synthesis duration^23^. Studies have indicated that the UV-Vis absorption patterns of AgNPs, synthesized using various plants such as *Entada spiralis*^36^ and *Cydonia oblonga*^37^ exhibit variations within the 400–500 nm range, contingent upon temperature conditions. The present study also observed the UV-Vis absorption peaks at approximately 450 nm, aligning with these reported findings. It was also determined that methanol and water used in the extraction also caused different UV absorption. AgNPs synthesized from methanol extracts formed sharper edge peaks in UV-Vis analyses. These results support that during the reaction, free silver ions are reduced by interacting with the substances in the plant extract and transformed into AgNPs^35,36^. The pH of the reaction plays a pivotal role in influencing the synthesis of NPs, particularly in terms of regulating their size and morphology. Actually, pH-dependent changes in the shape, size, and stability of NPs have been reported in previous studies^8,38^. The ability of acids and bases to change the charges on biomolecules affects the binding of silver ions to biomolecules, resulting in the reduction of silver ions to nanosilver. Generally, the best results have been achieved under alkaline pH conditions^39,40^. Sathishkumar et al. indicated that large NPs were formed at lower pH, where highly dispersed, small NPs were formed at high pH. With further decrease in pH, aggregation overcomes nucleation to form large particles. However, at higher pH conditions supported by fast reaction, more functional groups are available to bind to Ag, leading to small-sized NP synthesizing indicating the formation of stable and highly homogeneous NPs^40^. In this study, when examined the UV-Vis spectra, absorbances of AgNPs synthesized in pH 4 were quite low, but AgNPs synthesized in pH 7 and pH 10 showed more intense absorbances and the peaks were consistent with the absorbances of typical AgNPs between 300 and 600 nm. Alqadi et al.^47^ also reported that the pH of reaction changed the UV-Vis spectra of AgNPs and the top points of the peaks observed in the UV spectrum of increasing pH shifted towards smaller wavelengths. Zia et al.^42^ found that NP synthesis did not occur when pH was adjusted to 3, but specific UV absorption of NPs was obtained between pH 4 and 9. The green synthesis of AgNPs is also affected by the temperature conditions^3^. Temperature, a very important parameter in increasing the activation energy to carry out a reaction, is thought to directly influence physicochemical changes such as increased ion release and oxidation that prompt morphological changes in the system leading to particle aggregation^40^. Previous studies showed a general increasing trend in NP synthesis with the rise of temperature^41^. Zia et al.^42^ reported that in the reactions carried out at 50, 70 and 90 °C, the UV absorption at 90 °C was low, while the peaks obtained at 50 and 70 °C were more pronounced. In other words, it was determined that the wavelength of the top point of the peak decreased as the temperature increased. In this study, it was observed that the top point of the peak was more pronounced in the reaction at 60 °C in accordance with previous studies.

FT-IR analysis was carried out to clearly reveal the presence of diverse functional groups in the synthesized AgNPs, which play a pivotal role in the biological reduction of AgNO_3_ to AgNPs using the leaf extract. The FT-IR spectrum confirms the presence of -NH and -OH groups in AgNPs synthesized from grapevine extract rich in secondary metabolites, evidenced by the peaks at 3200–3500 cm^− 1^. Similarly, the peak at 3000–2800 cm^− 1^ indicates the presence of C-H asymmetric methylene stretching. There is also another characteristic peak value corresponding to the 1800–1600 cm^− 1^ peak indicating the presence of aromatic C=C groups. Terpenoids, flavones and polysaccharides show the presence of –C–O groups corresponding to a peak of 1450–1350 cm^− 1^. The FT-IR spectra clearly show that AgNPs are stabilized by biomolecules as polyphenolic moieties in the plant extract^43^. The carbonyl (C=O) functional group and the hydroxyl (OH) groups formed by the reduction of this group in the grapevine extract have the potential to interact strongly with metal NPs and form bonds. AgNPs are surrounded by polar carbonyl and hydroxyl functional groups in the structure of amino acids and proteins in the plant extract, thus the potential of the metal particles to come together and form agglomeration decreases and their stability in the solution environment increases^14,44^. In this study, it was determined that the FT-IR spectra of AgNPs synthesized using extractions with methanol under the same reaction conditions were more prominent and contained more functional groups. The reason for this is thought to be the presence of more functional groups to be reduced by silver ions due to the higher solvent effect of methanol than water. FT-IR spectra of the AgNPs synthesized with methanol extracts showed high similarity, but there were significant differences among the AgNPs synthesized with water extracts. In the literature review, there was no study conducted on the differences in the FT-IR spectra of synthesized AgNPs due to different extraction methods, temperature and pH of the reaction.

In the study, XRD analysis was also carried out to determine the degree of crystallinity and particle size of the AgNPs. As a result of comparisons with databases and previous studies, synthesized AgNPs were generally in a spherical form with a cubic structure at the center, rarely multi-edged^36,42^. Especially in NP7, NP12, NP18 and NP19, triangular, cube, and multi-edged particles are also available. Various factors such as temperature and pH during synthesis are known to affect the psycho-chemical properties of AgNPs, leading to the production of different behaviors and applications^45,46^. Research conducted by Manosalva et al.^15^ and Miranda et al.^45^ has revealed that pH represents a crucial reaction parameter with direct implications on particle size distribution, agglomeration tendencies, and morphology. Also in this study, the pH variation produced AgNPs of different sizes and shapes. AgNPs with triangular, cube and multi-sided shapes were obtained at pH 4 and 10. At pH 7, peaks of larger size were formed. It was determined that 20 min at 60 °C + 4 h at room temperature with methanol and 20 min at 60 °C + 4 h at room temperature caused more intense peaks. This indicates that there are more ions to be reduced by silver ions in the methanol extract.

HR-TEM imaging showed that the AgNP sizes varied depending on extraction and reaction conditions, with only certain NPs (NP10, NP16, NP22, and NP23) forming small groups and aggregating. Consistent with our study, it has been reported that AgNPs synthesized also in *Entada spiralis*^36^, quince^37^, *Cydonia oblong*^42^ plants were not uniformly aggregated but had a spherical structure with an average size ranging from 3 to 50 nm, which can approach each other in small groups. In our study, AgNPs synthesized from methanol extracts were found to be less aggregated at pH 7 and well homogenized during TEM. AgNPs were found to be in small groups attached to each other with electron clouds at pH 4 and 10. Alqadi et al.^47^ reported that increasing pH values caused AgNPs to decrease in size and become more spherical in shape. In this study, it was observed that, as the pH increased, NPs were generally found to be smaller in size and had a more spherical structure, but as previously mentioned^9^, these trends varied depending on the extraction and reaction temperature.

DLS, also known as photon correlation spectroscopy or semi-elastic light scattering^48^, is one of the methods used to determine the size of NPs in colloidal suspension at the nanoscale based on Brownian motion. In DLS analysis, a beam is directed toward the particles, and the system records the variations in intensity within the scattered beam. According to the DLS analysis, the average size of the synthesized AgNPs was found to vary between 8 and 60 nm. These results are very similar to the particle sizes calculated by the Debye-Scherrer equation. In the study, NP particle size varied depending on extraction and reaction conditions. Armendariz et al.^49^ also stated that pH plays an important role in the synthesis of NPs from plants by green synthesis and that the pH value of the solution medium affects the size and shape of the synthesized NPs. It has also been reported in previous studies that increasing pH decreases the size of AgNPs and causes their shapes to become more spherical^47,50^. This is due to the formation of nucleation centers that increase with the rise in pH. As the nucleation center increases, the reduction of metallic ion to metal NPs also increases. In the same case, the pH of the solution affects the activity of functional groups in the plant extract and also the reduction rate of the metal salt^51^. As Satishkumar et al.^52^ pointed out, pH plays an important role in controlling the size of AgNPs during synthesis. At higher pH, small, stable NPs are formed due to electrostatic repulsion, while at lower pH, large, aggregated NPs are formed. Again, rising in temperature leads to a decrease in the size of AgNPs and an increase in polydispersity. The decrease in particle size with temperature is because most silver ions are consumed in the nuclei formation due to the increased reaction rate and temperature, and the secondary reduction process is stopped on the surface of already formed nuclei^53^. In this context, optimization of pH and temperature reduce the size of particles with longer stability^54^.

The zeta potential, also known as electrokinetic potential, with values between + 100 mV and − 100 mV, is a stability measure of the charge of NPs and is responsible for the interactions between NPs in suspension^55^. Zeta potential is an important parameter for the determination of surface charge and stability of AgNPs synthesized in aqueous solution. Zeta potential values higher than + 30 mV or lower than − 30 mV are considered as indicators of a stable system^56^. Lower dispersions lead to coagulation or agglomeration due to Van der Waals interparticle attraction^57^. In this study, AgNPs generally have a zeta potential greater than + 30 mV and less than − 30 mV and are in a stable structure. However, AgNPs NP7, NP10, NP16, NP22 and NP23 were found to be less stable with zeta potential values of − 29, − 20, − 18, − 25 and − 29 mV, respectively, and aggregated by adhering to each other. These results also coincide with HR-TEM microscopy.

Numerous reports highlight the favorable aspects of applying NPs in plant tissue culture. AgNPs are the most commonly used NPs due to their potential to significantly enhance cell growth, biomass production, and secondary metabolite production in plants, along with their antimicrobial effects compared to other metal particles^4,18,19,58^. AgNP treatments were shown to promote callus proliferation, growth kinetics, and fresh and dry biomass accumulation in callus cultures^17,18^. In the present study, the highest values in cell growth were detected in the cells treated with NP17. This result indicated that the synthesis carried out in room temperature at pH 7 with methanol extract positively affected cell growth. AgNPs can boost the activation of the enzymatic pathway responsible for increasing the accumulation of phytochemicals^58^. AgNP treatments have been shown to increase the synthesis of stress enzymes and phenolic compounds in plants^19,59^. In the present study, the amounts of phenolic compounds varied depending on the method used to acquire the NPs. The highest total phenolic compound, *trans-*resveratrol, catechin and epicatechin amounts were obtained from NP11 and NP5. For the production of these phenolic compounds, the use of aqueous or methanol extraction to obtain AgNPs was not significant. However, especially AgNPs synthesized at room temperature and pH 7 were quite effective in the production of these compounds. This is thought to be a result of the extremely small particle sizes of NP11 and NP5. When the size of NPs decreases, they become more reactive and their biological activity increases due to the rise in surface/volume ratio^1^. In the study, the production of phenolic compounds except *o*-coumaric acid and cinnamic acid, which yielded the highest values in control cells, was generally positively influenced by the AgNP applications. Especially AgNPs synthesized in room temperature and pH 7 using E1 and E2 extracts were found to be the most favorouble AgNPs for increasing the phenolic accumulation. The increase in the amounts of phenolic compounds with AgNP treatment may be because of silver ions on the metabolic flow between various phenolic acids^16^. As indicated in previous studies showing that silver ions can also act as an elicitor^17,19,22^, silver ions supplied in the form of AgNO_3_ inhibit ethylene synthesis while increasing the accumulation of many secondary metabolites, including phytoalexins, in plants^60^. In our study, *trans-*resveratrol was the quantitatively most predominant phenolic compound as a result of HPLC analysis. It is known that grapevine leaves are very rich in *trans-*resveratrol among the phenolic compounds examined^61,62^. Teszlak et al.^63^ investigated the effects of titanium dioxide NPs applied in a spray form to grapevine leaves on photosynthetic activity and the flavonol profile of grapes. Almagro et al.^27^ examined the effects of magnetic NPs coated with cyclodextrin polymers on resveratrol amounts by applying them to the callus of the Monastrell grape variety. This goes to show that there is no study has examined the effects of AgNPs on the accumulation of neither *trans-*resveratrol nor other phenolic compounds in grapevine cell suspension cultures. However, our findings agree with earlier studies on other plants^64,65^. Vecerova et al.^66^ reported that the increase in the amount of secondary metabolites, amino acids and vitamins as a result of NP applications was due to the increased production of reactive oxygen species. The initial response of plants to NPs is associated with elevated ROS levels, cytoplasmic Ca2+ and mitogen-activated protein kinase (MAPK) cascades, like abiotic stresses^67^. These results indicate that MAPK genes play a role in promoting the upregulation of genes associated with the synthesis of secondary metabolites.

## Conclusion

The results show that characteristic properties of AgNPs altered significantly depending on the solvent used in extraction, extraction method, pH and conditions of the reaction solution during synthesis. Moreover, the production of phenolic compounds in cell suspension cultures was influenced by AgNPs derived from different synthesis methods. We optimized and verified the parameters that had a significant impact on the synthesis and stability of AgNPs. The NP11 and NP5 AgNP treatments resulted in the highest accumulation of total phenolic compounds, *trans-*resveratrol, catechin and epicatechin in the cells. The pH 7 and maintenance at room temperature for 4 h played crucial roles in the accumulation of these compounds. In the extraction procedure, the use of methanol or water did not affect the production of phenolic compounds while the extraction procedure had a significant impact on this content. It was found that the extraction procedure involving boiling steps (E1 and E2) is more effective. These results indicate that AgNPs can be a successful production method for the in vitro production of phenolic compounds in grapevines, provided that an appropriate synthesis method is chosen. To the best of our knowledge, our study is the first to analyze the effects of green synthesized AgNPs on in vitro phenolic compound production in grapevine. Further research is required to gain a better understanding of the interactions between NPs and the secondary metabolism of plants and to enhance the utilization of NPs in regulating the biosynthesis of metabolites.

## Electronic supplementary material

Below is the link to the electronic supplementary material.

Supplementary Material 1

## Data Availability

The datasets generated during and/or analysed during the current study are available from the corresponding author on reasonable request.
