# Supplementary figures and images for: Synthesis methods impact silver nanoparticle properties and phenolic compound production in grapevine cell cultures
Source: Sci Rep. 2025 Mar 5;15:7667. doi: 10.1038/s41598-025-85545-7 (PMC11882897; doi:10.1038/s41598-025-85545-7)

**Supplementary Material.** Zeta potentials of different 24 NPs


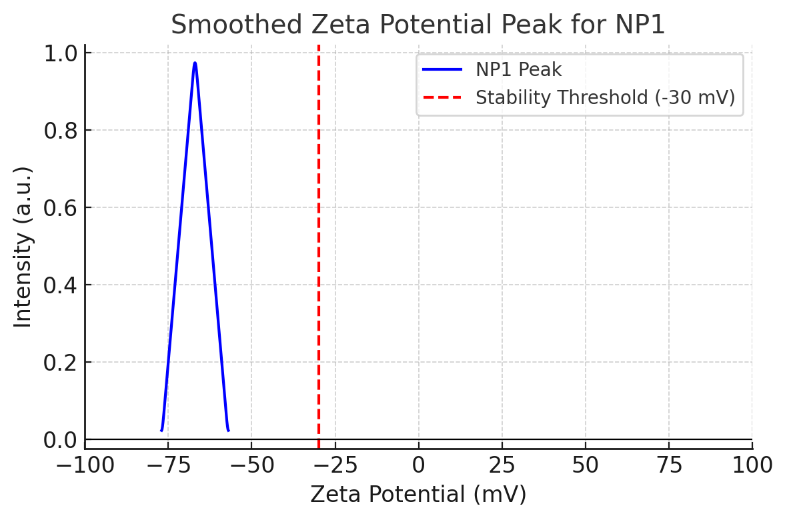

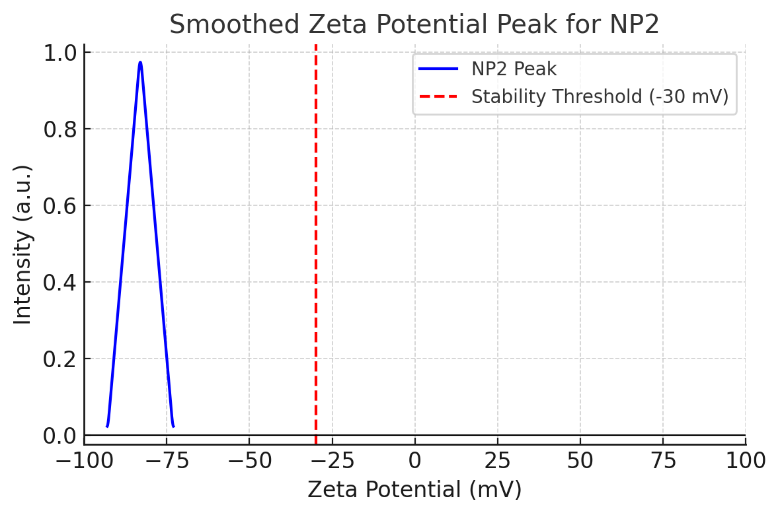

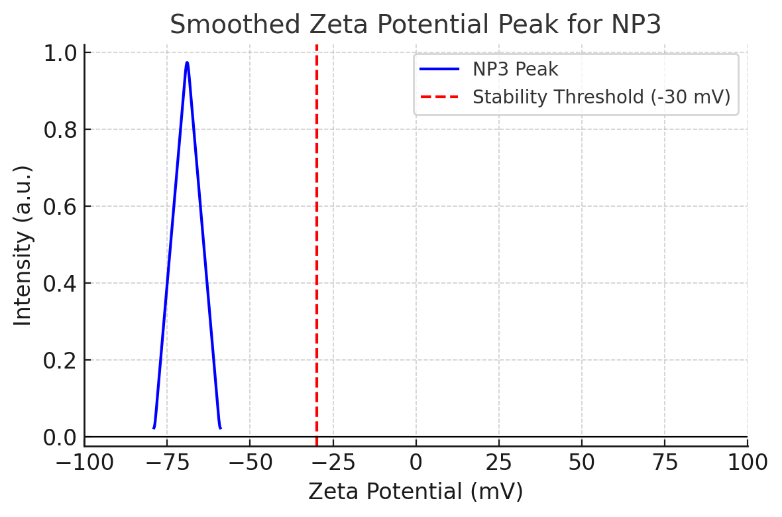

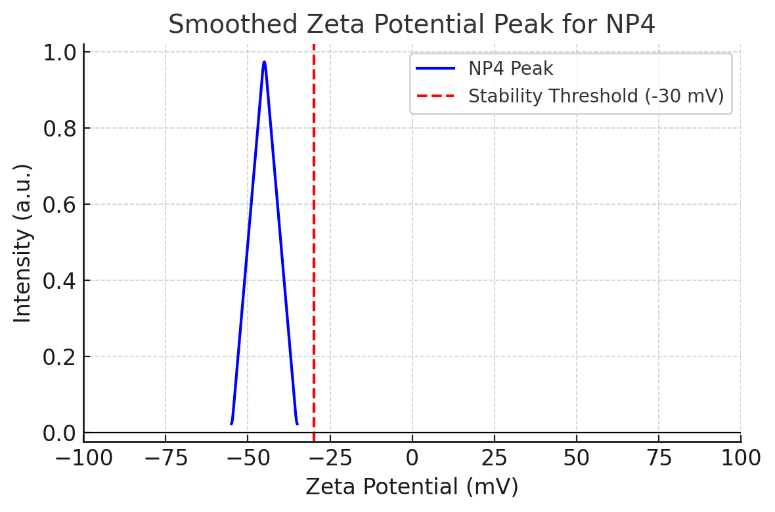

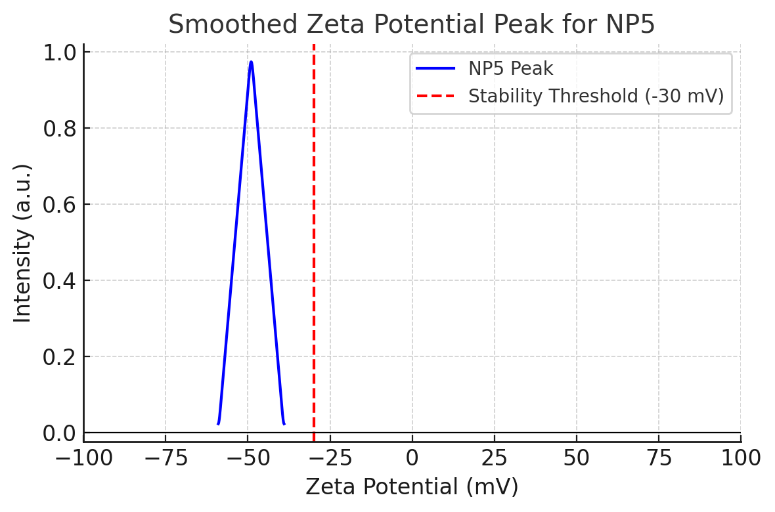

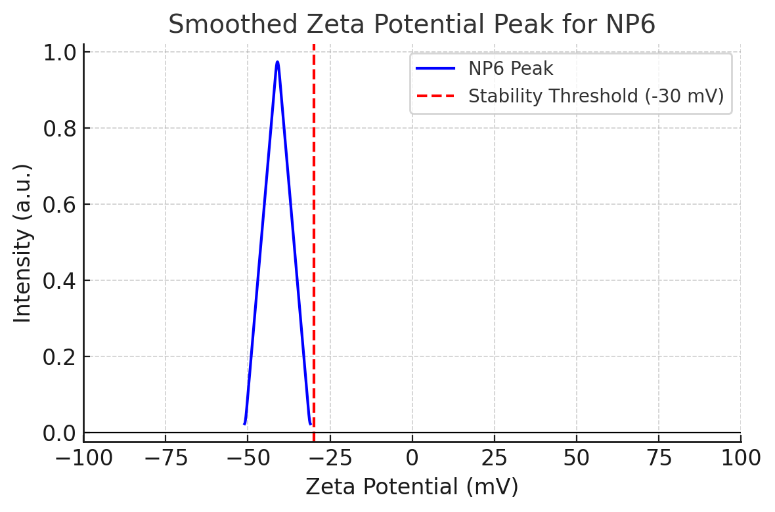

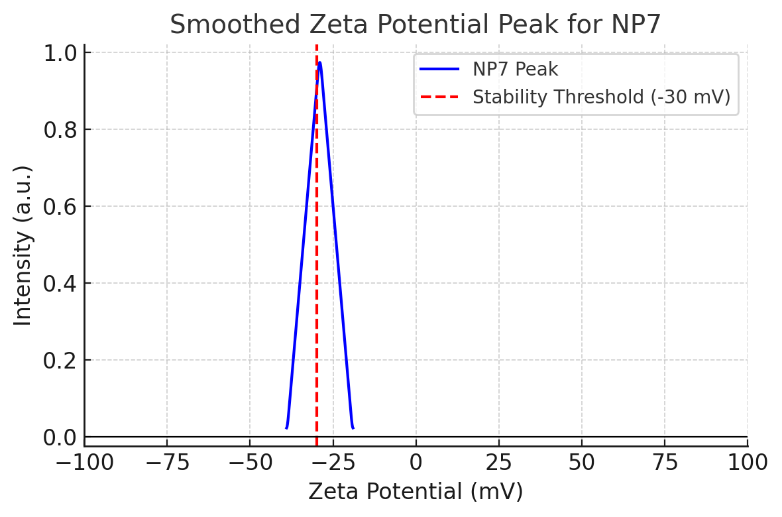

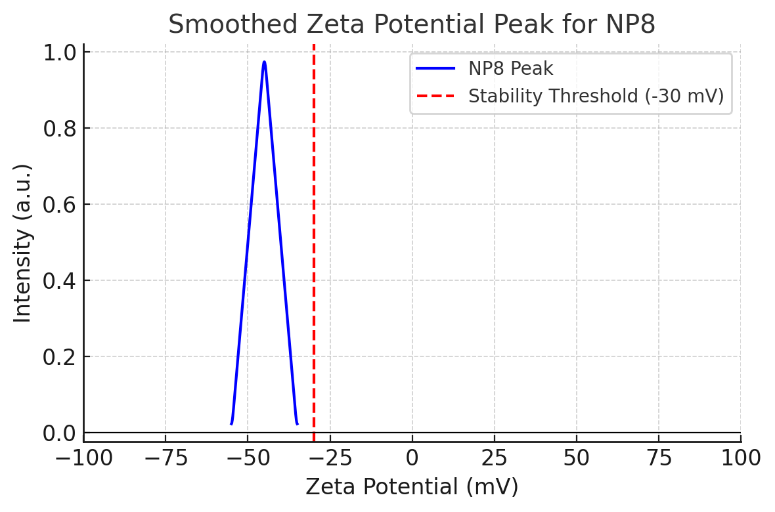

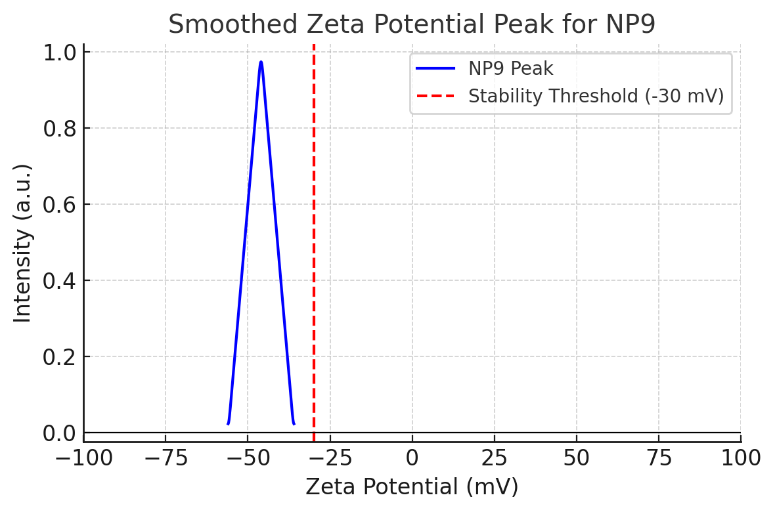

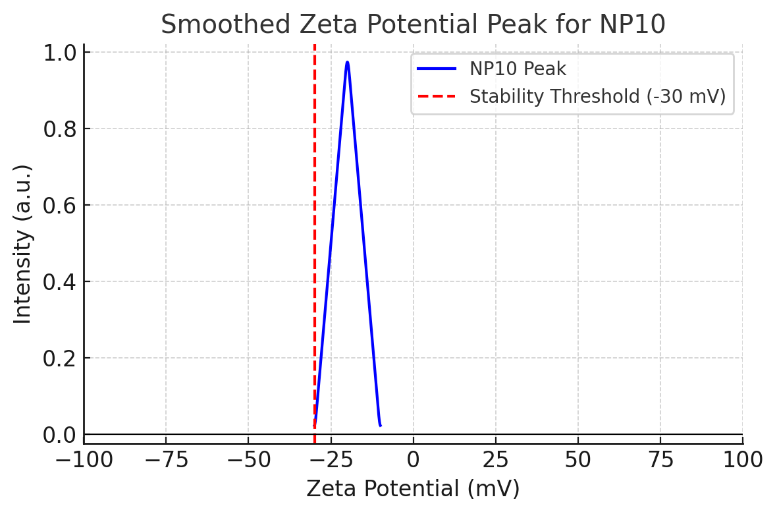

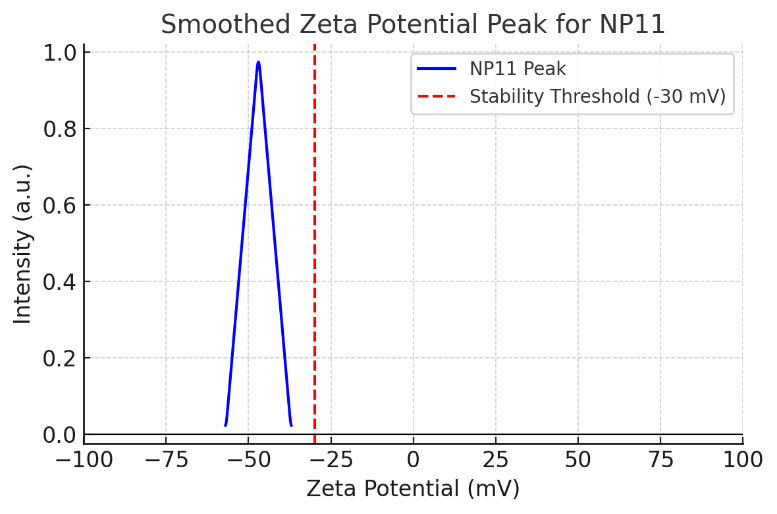

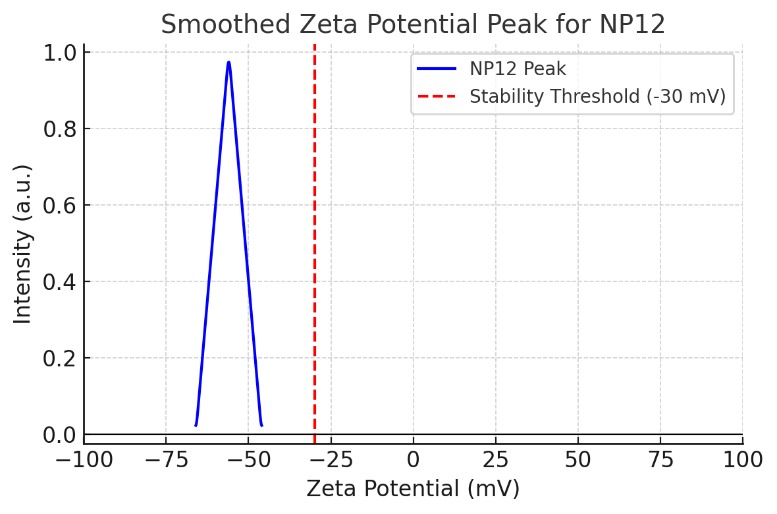

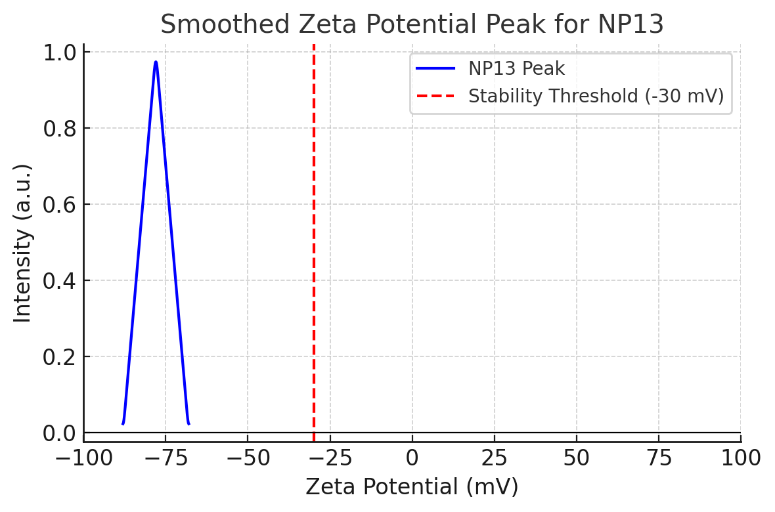

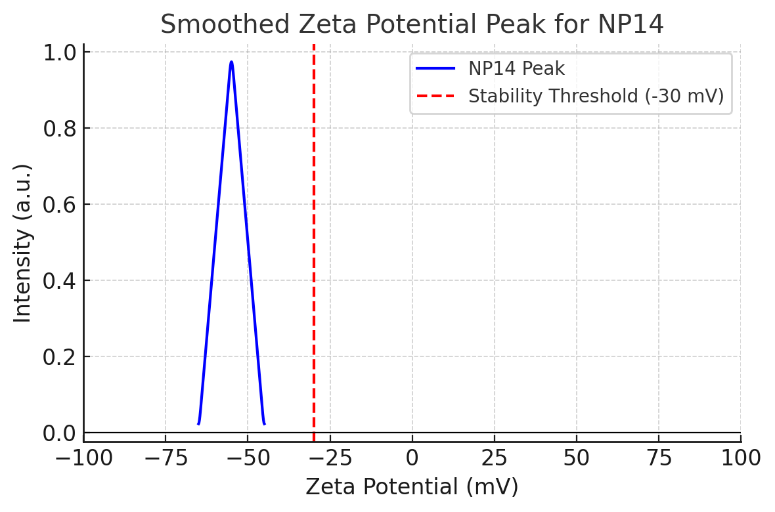

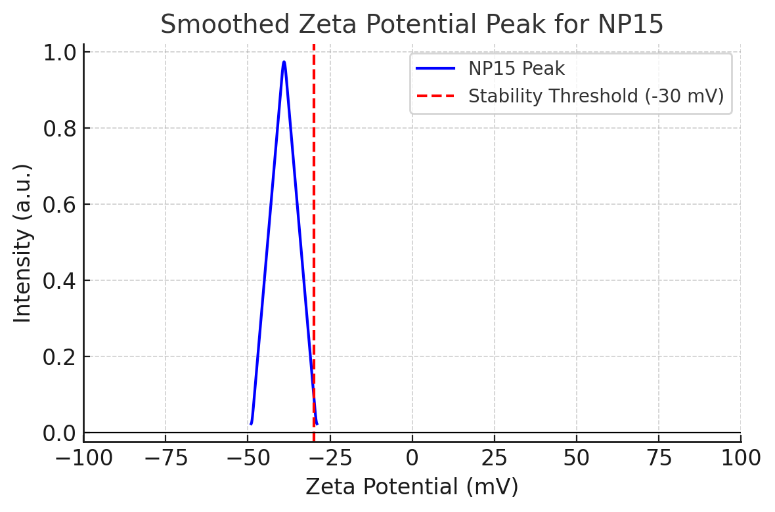

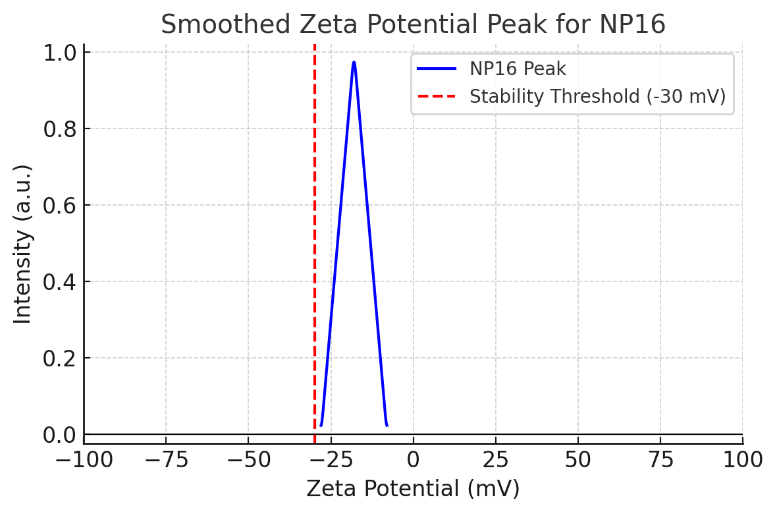

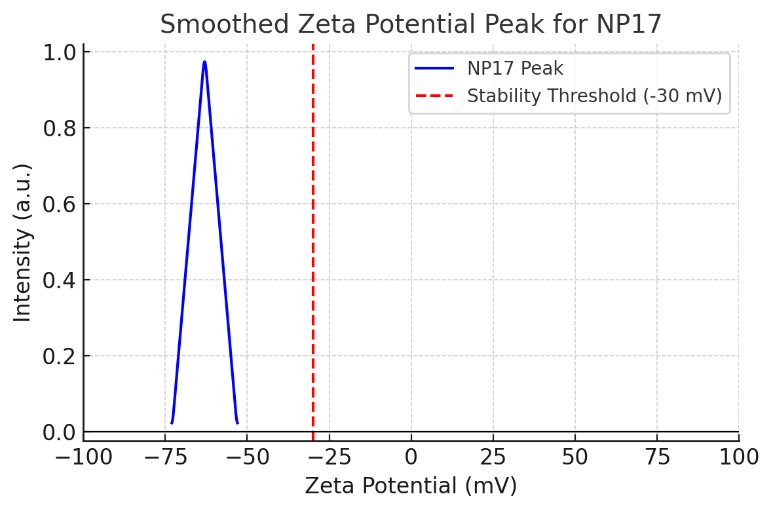

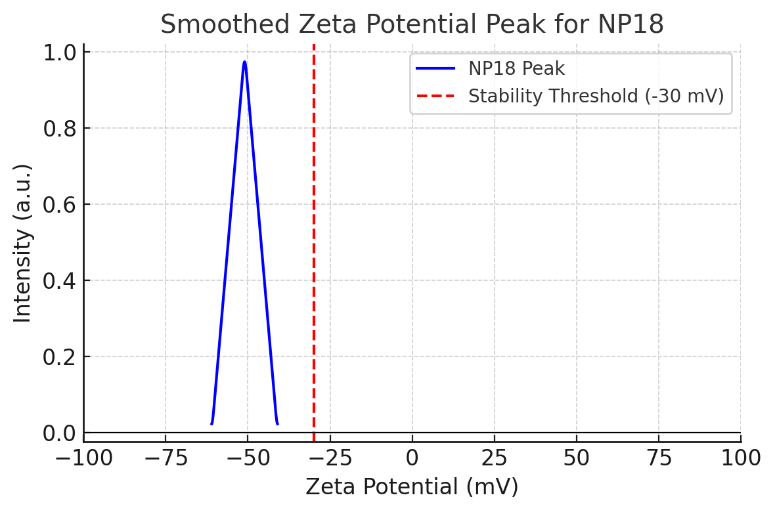

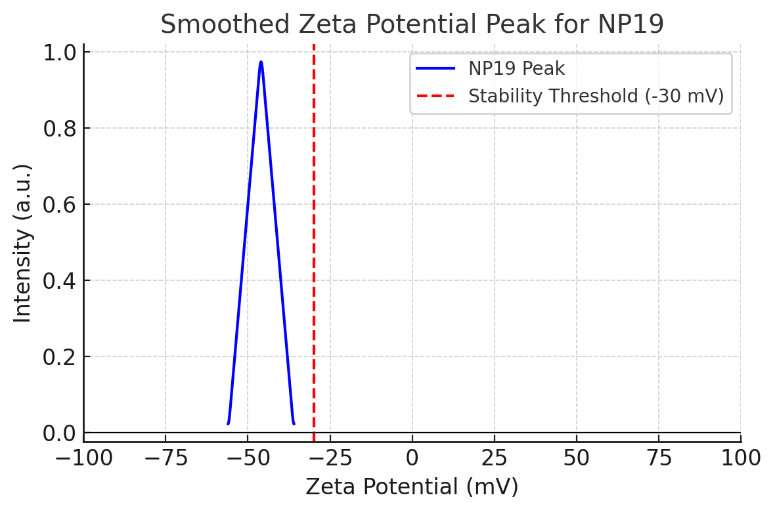

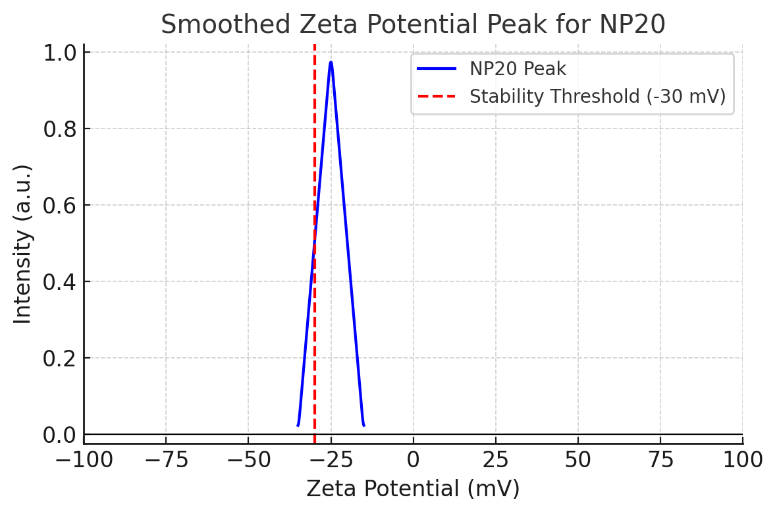

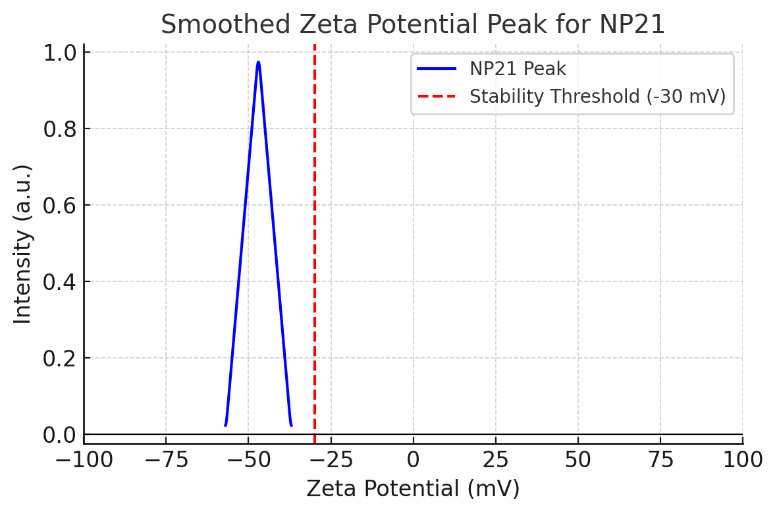

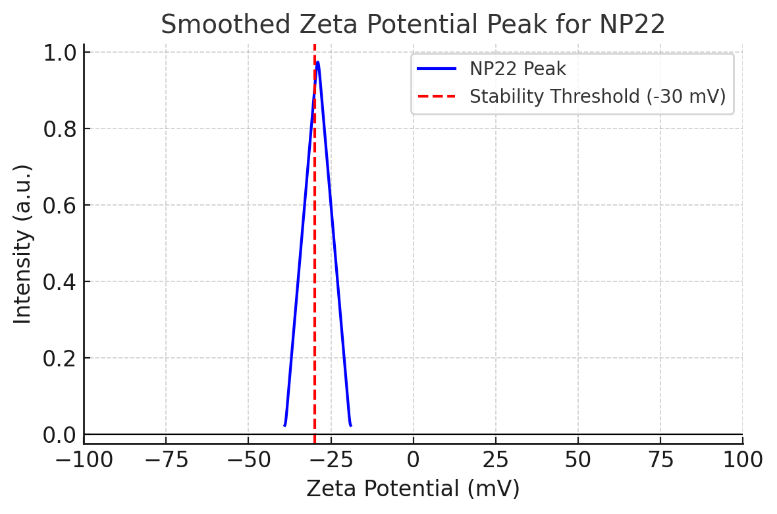

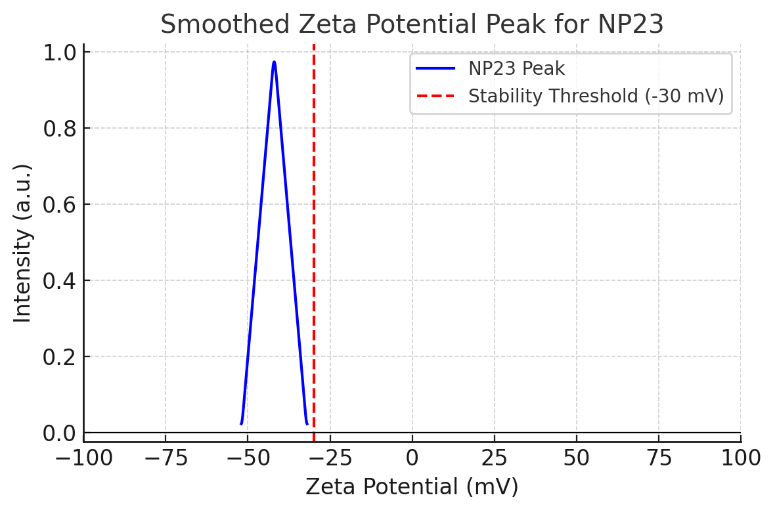

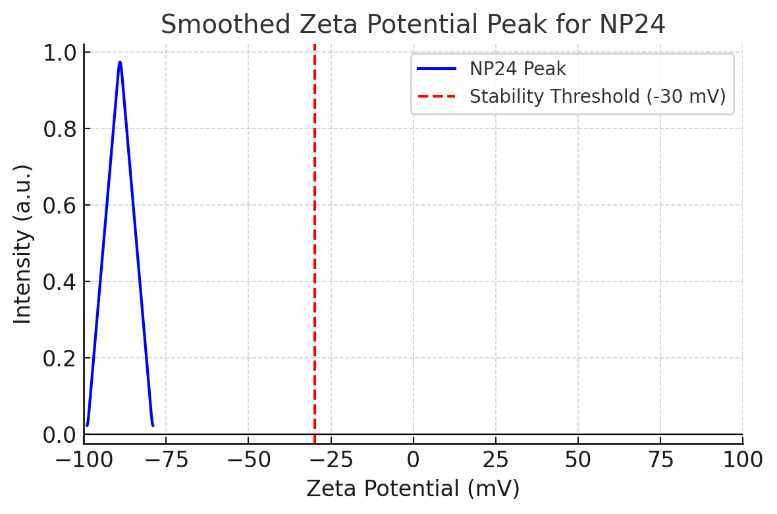

Supplement: Supplementary file 1 — Supplementary Material 1 [file 41598_2025_85545_MOESM1_ESM.docx]
